# Proline/serine-rich coiled-coil 1 alleviates atherosclerosis via remodeling tryptophan metabolism mediated by *Akkermansia muciniphila*

**DOI:** 10.1038/s12276-026-01668-5

**Published:** 2026-03-06

**Authors:** Qiao Wu, Kexin Hu, Qianqian Wang, Tiantian Luo, Lu Hu, Jichen Liu, Danfeng Zou, Jing Hu, Zhigang Guo

**Affiliations:** 1 Department of Cardiology, Huiqiao Medical Center, Nanfang Hospital, Southern Medical University, Guangzhou, China; 2 Department of Cardiology, Sichuan Provincial People’s Hospital, School of Medicine, University of Electronic Science and Technology of China, Chengdu, China; 3 Department of Cardiology, State Key Laboratory of Organ Failure Research, Nanfang Hospital, Southern Medical University, Guangzhou, China; 4 Huiqiao Medical Center, Nanfang Hospital, Southern Medical University, Guangzhou, China; 5 Department of Cardiology, Jiangxi Provincial People’s Hospital, The First Affiliated Hospital of Nanchang Medical College, Nanchang, China

**Keywords:** Atherosclerosis, Apoptosis, Ubiquitylation, Metabolomics, Foam cells

## Abstract

Genome-wide association studies have implicated proline/serine-rich coiled-coil 1 (*PSRC1*) in coronary artery disease (CAD) pathogenesis. Our previous studies demonstrated that Psrc1 deficiency accelerates atherosclerosis via gut microbial dysbiosis, characterized by a substantial depletion of *Akkermansia muciniphila*. Recent studies implicate microbiome-dependent tryptophan metabolism as a novel checkpoint in atherosclerosis, with specific microbial taxa regulating metabolite-driven immune responses. The mechanism by which Psrc1 modulates atherosclerosis through *A. muciniphila* and its regulation of tryptophan metabolism remains unclear. Here *Psrc1* knockout mice exhibited reduced colonic mucin content, altered tryptophan metabolic enzyme expression and diminished levels of Trp metabolites including indoleacetic acid (IAA), with concomitant suppression of Ahr signaling in macrophages. In vivo analysis revealed that *Psrc1* knockout diminishes Ahr through *A. muciniphila*-dependent IAA depletion. In vitro experiments further uncovered that Psrc1 stabilizes Ahr protein via ubiquitin carboxyl terminal hydrolase L3 (Uchl3)-mediated deubiquitylation. In addition, we identified plasma IAA levels positively correlating with decreased *PSRC1* expression in peripheral blood mononuclear cells from patients with CAD. Furthermore, therapeutic restoration of a live *A. muciniphila–*IAA axis through oral supplementation reversed atherosclerosis in *Psrc1* knockout mice. Notably, oral IAA supplementation substantially ameliorated atherosclerosis in *Psrc1* knockout mice by suppressing plaque macrophage apoptosis. Crucially, co-administration of the Ahr antagonist CH-223191 abolished these benefits, confirming Ahr dependence. Our findings position PSRC1 as a critical regulator of the *A. muciniphila–*IAA–Ahr axis and nominate microbiome-targeted Ahr activation as a precision therapeutic strategy for patients with CAD with *PSRC1* loss-of-function variants.

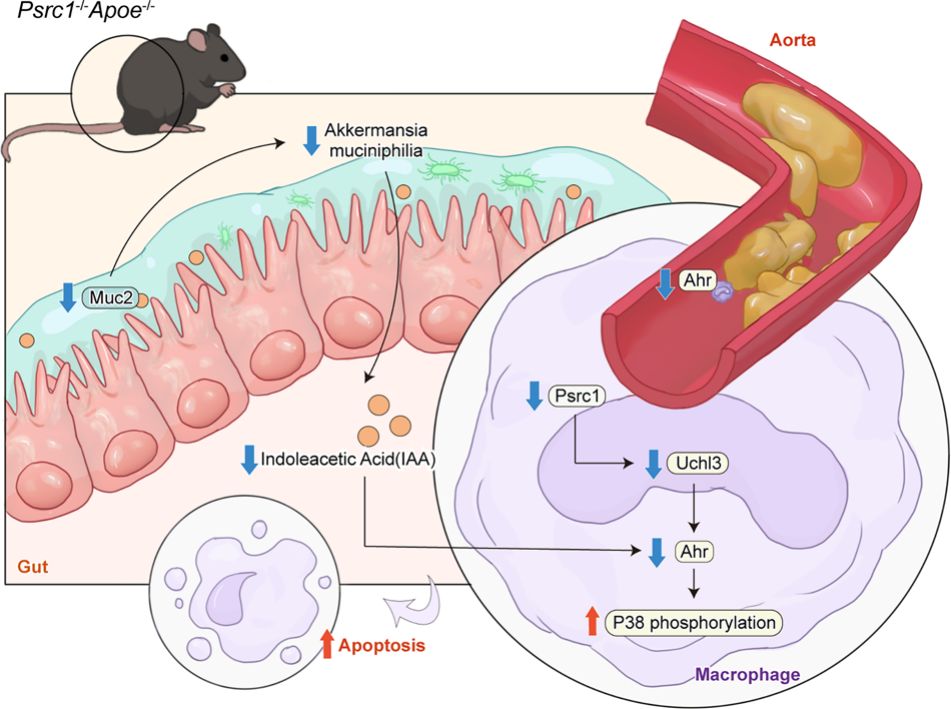

## A. muciniphila and PSRC1 link gut microbes to heart health

Atherosclerosis is a major cause of heart disease and stroke, involving the buildup of fats in arteries. Current treatments do not fully eliminate the risks so new approaches are needed. Here researchers explored the role of a protein called PSRC1 in atherosclerosis. They found that PSRC1 helps protect against artery damage by influencing gut bacteria and metabolism. They discovered that mice lacking PSRC1 had more artery damage and less of a beneficial gut bacterium called *Akkermansia muciniphila*. This bacterium helps maintain gut health and produces a compound called indole-3-acetic acid (IAA), which has protective effects against inflammation and cell death. The researchers concluded that PSRC1 supports heart health by maintaining healthy gut bacteria and producing protective compounds such as IAA. This study suggests that targeting gut bacteria and metabolism could be a promising strategy for treating heart disease in people with PSRC1 deficiencies.

This summary was initially drafted using artificial intelligence, then revised and fact-checked by the author.

## Introduction

Atherosclerosis is the principal pathological driver of coronary artery disease (CAD), myocardial infarction and ischemic stroke^1^. The hallmark of early atherosclerosis lies in the structural breakdown of arterial wall homeostasis, with oxidized low-density lipoproteins (ox-LDL) accumulation in the intima acting as a key instigator of both endothelial dysfunction and monocyte recruitment. Subsequently, both infiltrating macrophages and resident vascular smooth muscle cells internalize these modified lipoproteins via receptor-mediated endocytosis. This process drives their differentiation into lipid-laden foam cells, while concurrently triggering the secretion of proinflammatory cytokines that sustain and amplify vascular inflammation. The formation of an expanded necrotic core, predominantly arising from foam cell apoptosis, serves as a hallmark pathological feature in the progression of advanced and vulnerable atherosclerotic plaques^2^. Although current therapeutic strategies centered on lipid-lowering and anti-inflammatory mechanisms have yielded substantial clinical benefits, they remain inadequate in abrogating residual cardiovascular risks. This persistent clinical challenge highlights the imperative for novel therapeutic interventions that precisely target the complex and interconnected pathophysiological networks of atherosclerosis.

Proline/serine-rich coiled-coil 1 (*Psrc1*) is a regulator of spindle dynamics^3^. Genome-wide association studies have identified *PSRC1* as a susceptibility locus for myocardial infarction at a prespecified threshold, while also revealing its association with LDL-C levels^4,5^. Consistent with these findings, a recent multiomics analysis using the data in UK Biobank data further validated PSRC1 as a promising therapeutic target for atherosclerosis^6^. Building on this, our previous work demonstrated that *Psrc1* functions as an atherosclerosis-protective factor, exerting its beneficial effects partly through the regulation of gut microbiota composition and metabolic conversion to mitigate atherosclerosis^7,8^. Notably, accumulating evidence highlights the pivotal role of gut microbiota and dietary-derived bioactive nutrients in driving atherosclerosis pathogenesis^9,10^. The depletion of gut microbiota has been shown to accelerate atherosclerosis and the impact of the gut microbiota on atherosclerosis is tightly linked to dietary patterns^11,12^. Our previous study using *Apoe*^−/−^ mice with *Psrc1* knockout revealed profound intestinal flora dysbiosis, characterized by increased abundance of pro-atherogenic bacterial taxa and reduced levels of beneficial microbes. Among these changes, *Akkermansia* exhibited one of the most striking alterations in abundance^8^.

*Akkermansia muciniphila*, a representative bacterium of the phylum *Verrucomicrobiota*^13^, has emerged as a key modulator of host health and disease. Accumulating evidence indicates that reduced abundance of *A. muciniphila* correlates with the pathogenesis of multiple pathological conditions. Specifically, *A. muciniphila* has been demonstrated to attenuate atherosclerosis by restoring colonic barrier integrity, thereby mitigating systemic inflammatory responses triggered by endotoxemia^14^. *A. muciniphila* possesses the capacity to degrade and utilize the mucin as the source of carbon, nitrogen and energy, while the intestinal mucus layer in turn provides nutrients and attachment sites to support its colonization. In addition, *A. muciniphila* contributes to the restoration of mucus layer thickness and the maintenance of gut homeostasis. Notably, interventional studies have demonstrated that administration of living or pasteurized *A. muciniphila*, its constituent proteins (P9 and Amuc_1100), or derived metabolites can exert beneficial effects, encompassing the regulation of metabolic homeostasis, modulation of immune responses and suppression of excessive inflammation^14–17^. Given the established role of *A. muciniphila* in atherosclerosis protection and the link between Psrc1 deficiency, gut microbial dysbiosis and accelerated atherosclerotic progression, investigating the therapeutic potential of *A. muciniphila* supplementation in ameliorating Psrc1 deficiency-exacerbated atherosclerosis represents a compelling and necessary research direction.

Tryptophan (Trp), an essential amino acid, is catabolized in the host via the kynurenine, indole and serotonin pathways^18^. Its diverse metabolites, including kynurenic acid (KYNA), quinolinic acid (QA), indole, indole-3-propionic acid (IPA), indoleacetic acid (IAA), indole-3-lactic (ILA) and serotonin, exert pleiotropic regulatory effects on key biological process, encompassing inflammation, immune responses and glucose and lipid metabolism^19–24^. Accumulating evidence indicates that Trp metabolites confer protective effects against metabolic and inflammatory disorders such as obesity, nonalcoholic fatty liver disease, colitis and atherosclerosis^20,21,25,26^. By contrast, other Trp-derived metabolites have been implicated in the pathogenesis of kidney injury, cardiac hypertrophy or neurotoxicity^27–29^, highlighting the dual functional nature of Trp metabolism.

Notably, the indole pathway is predominantly mediated by gut microbiota-derived enzymes and its derivative indole metabolites serve as aryl hydrocarbon receptor (Ahr) ligands^30^. It has been reported that supplementation with indole derivatives mitigates vascular inflammation and attenuates atherosclerotic lesion progression^31^. Furthermore, Trp supplementation has been shown to partially reverse antibiotic-induced exacerbation of atherosclerosis, underscoring the complex crosstalk between host Trp metabolism and the intestinal microbiota in modulating atherosclerotic pathogenesis^32^.

Intriguingly, dietary Trp supplementation promotes the growth of *A. muciniphila*, while in vitro studies have confirmed that *A. muciniphila* can metabolize Trp to indole, IAA and ILA^33^. In addition, patients with ulcerative colitis exhibit notably reduced *A. muciniphila* abundance and circulating IAA levels. Importantly, administration of live or pasteurized *A. muciniphila*, as well as its outer membrane protein Amuc_1100, effectively rescues dextran sulfate sodium-induced colitis in mice by restoring IAA production^34^. However, the functional role and underlying mechanisms of IAA in atherosclerosis remain poorly defined. Specifically, whether and how IAA modulates atherosclerotic progression exacerbated by Psrc1 deficiency represents a critical knowledge gap warranting further investigation.

Here, we hypothesized that Psrc1 deficiency drives *A. muciniphila*-mediated reprogramming of Trp metabolism, ultimately exacerbating atherosclerosis. Herein, we first identified the mechanism underlying *A. muciniphila* decline in Psrc1 deficiency mice and identified IAA as the key bioactive mediator through which *A. muciniphila* modulates atherosclerosis. Subsequently, we investigated the protective roles and underlying mechanisms of *A. muciniphila* and IAA in alleviating Psrc1 deficiency-induced atherosclerosis using in vivo and in vitro approaches. This work unravels a novel regulatory axis wherein Psrc1 mitigates atherogenesis by orchestrating *A. muciniphila*-dependent Trp metabolic reprogramming, providing new insights into the crosstalk between host genetics, gut microbiota and amino acid metabolism in atherosclerosis. Importantly, our findings establish a preventative and therapeutical strategies tailored to patients with CAD harboring PSRC1 loss-of-function variants. Furthermore, the identification of this specific Trp metabolic pathway offers a potential framework for precision dietary interventions that could be translated into clinical practice to mitigate atherosclerotic risk.

## Material and methods

### Mice and experimental design

All the animal experiments in this study were approved by the Animal Experiment Committee of Nanfang Hospital at Southern Medical University. C57BL/6J mice were purchased from Guangdong Medical Laboratory Animal Center. *Apoe*^−/−^ mice were obtained from Viewsolid Biotech Co. Ltd., which were called *Psrc1*^+/+^*Apoe*^−/−^, while *Psrc1*^−/−^*Apoe*^−/−^ mice were generated in the background of *Apoe*^−^^/−^ using the CRISPR–Cas9 system via deleting exon 4, which is essential for the function of Psrc1 protein, as in our previous study^8,35^. Offspring from the parental lineages served as the mice model in the current study. The experimental mice were acclimatized and fed with the chow diet in the specific pathogen-free environment at the Experimental Animal Research Center of Nanfang Hospital in Southern Medical University. Eight-week-old male *Psrc1*^+/+^*Apoe*^−/−^ and *Psrc1*^−/−^*Apoe*^−/−^ mice were randomly assigned to distinct treatment groups prior to initiation of a 12-week atherogenic high-fat diet (HFD) feeding regimen. The subsequent experimental interventions were implemented in accordance with the specific design of each respective assay as detailed below:

#### Experiment 1

For the analysis of the effect and mechanism of *Psrc1* knockout on Trp metabolism throughout the progression of atherogenesis, 8-week-old male *Psrc1*^+/+^*Apoe*^−/−^ and *Psrc1*^−/−^*Apoe*^−/−^ mice were fed a HFD (Guangdong Medical Laboratory Animal Center) for 12 weeks. Biological samples including blood, peritoneal macrophages, jejunum, ileum, colon, aorta and aortic root were systematically collected following anesthesia with isoflurane.

#### Experiment 2

For analysis of the effect of *A. muciniphila* supplementation on *Psrc1* deficiency-mediated atherogenesis and reduction of IAA, 8-week-old male *Psrc1*^+/+^*Apoe*^−/−^ and *Psrc1*^−/−^*Apoe*^−/−^ mice were fed a HFD for 12 weeks. At week 6 of HFD initiation, mice were pretreated with a broad-spectrum antibiotic cocktail (vancomycin 0.5 g/l, neomycin 1 g/l, metronidazole 1 g/l and ampicillin 1 g/l) for 14 consecutive days. Following antibiotic clearance, *Psrc1*^+/+^*Apoe*^−/−^ and *Psrc1*^−/−^*Apoe*^−/−^ mice were randomly assigned to receive daily oral gavage of either 1 × 10⁹ c.f.u./ml live *A. muciniphila*, heat-killed *A. muciniphila* (treated at 121 °C for 15 min) or an equivalent volume of phosphate-buffered saline (PBS) as a control, with the intervention lasting for 28 days. One group received oral gavage with live *A. muciniphila*, while another group received equivalent *A. muciniphila* heated at 121 °C for 15 min. Biological specimens including blood, peritoneal macrophages, aorta and aortic root were systematically collected following anesthesia with isoflurane.

#### Experiment 3

For the analysis of the effect and mechanism of IAA supplementation on *Psrc1* deficiency-mediated atherogenesis, 8-week-old male *Psrc1*^+/+^*Apoe*^−^^/−^ and *Psrc1*^−/−^*Apoe*^−/−^ mice were fed a HFD for 12 weeks. Starting from week 8 of HFD feeding, the intervention was conducted for a consecutive 28 days. *Psrc1*^+/+^*Apoe*^−/−^ were randomly assigned to receive daily oral gavage of vehicle, 50 mg/kg/day IAA (MedChemExpress) or 15 mg/kg/day atorvastatin (Selleck Chemicals) as a positive control. In parallel, *Psrc1*^−/−^*Apoe*^−/−^ were randomly assigned to receive equivalent vehicle, IAA monotherapy or IAA combined with 10 mg/kg/day Ahr inhibitor CH-223191 (Selleck Chemicals). For the combined treatment group, CH-223191 was administered via oral gavage 2 h before IAA supplementation. Biological specimens including blood, aorta and aortic root were systematically collected following anesthesia with isoflurane.

#### Experiment 4

For the analysis of the toxic effects of graded doses of IAA in *Apoe*^−/−^ mice, 16-week-old male *Apoe*^−^^/−^ mice were orally administered vehicle, 10, 50, 250 or 500 mg/kg IAA daily for 4 weeks. Blood samples and heart tissues were collected after anesthesia.

### Patients

The procedures in this study were approved by the Ethics Committee of Nanfang Hospital, Southern Medical University, China (approval number NFEC-2019-185). Blood samples from 26 patients without CAD 77 patients with CAD were collected in the cardiovascular department of our hospital. The patients with CAD were diagnosed when coronary angiography revealed ≥50% stenosis in any major coronary artery or in its major branch. The patients without CAD were hospitalized for chest pain but coronary angiography indicated <50% stenosis in all major coronary artery and the branches^36^. Patients with heart failure, severe liver and renal dysfunction, infectious diseases, autoimmune diseases, malignancy or treated with antibiotics were excluded in this study. This study was performed according to the guidelines of Declaration of Helsinki and all the participants provided informed consent. The baseline clinical characteristics of the enrolled participants are presented in Supplementary Table 1.

### Assessment of aortic atherosclerotic lesions

For analysis of the en face plaque area percentage of aorta, the aorta in mice was dissected, fixed with 4% paraformaldehyde (G1101, Servicebio) for 48 h and then stained with Oil red O (ORO; Yuanye Bio-Technology). For the analysis of the content of plaques in the aortic root, the heart of mice was dissected, fixed with 4% paraformaldehyde and embedded with OCT (Sakura) compound or paraffin. Sequential cross-sections of aortic roots were sliced, then the tissue fixed-frozen or paraffin sections were stained with hemotoxylin and eosin (HE) to detect the lesion area and necrotic cores in plaques, and the tissue fixed-frozen sections were stained with ORO to detect lipids content in plaques and Masson’s trichrome to detect the collagen content. The macrophage and smooth muscle cells in plaques were detected via immunofluorescence staining with antibodies against CD68 (97778S, CST) and α-SMA (19245S, CST), respectively. For the analysis of Psrc1 in macrophages and smooth muscle cells or Ahr in macrophages, tissue slides were stained by co-immunofluorescence with Psrc1, CD68 and α-SMA or Ahr and CD68. To detect apoptotic macrophages in plaques, tissue slides were stained by co-immunofluorescence with TUNEL (G1506, Servicebio) and CD68. The quantification was analyzed via Image J software.

### Immunofluorescence staining

Tissue slides were fixed with 4% paraformaldehyde and cells in a confocal dish with methyl alcohol for 30 min, washed in PBS for 5 min and incubated with 5% BSA (w/v) in PBS for 1 h at room temperature. Tissue slides or cells were incubated with primary antibodies, such as anti-Psrc1 (GTX128047, GeneTex), anti-CD68, anti-α-SMA or anti-Ahr (28727-1-AP, Proteintech), overnight in 4 °C environment and incubated with Alex Fluor 488, Cy3 or Cy5-conjugated secondary antibodies (SeraCare Life Sciences) for 1.5 h in a dark room. Slides or cells were then incubated with 4,6-diamidino-2-phenylindole (DAPI) (D9542, Sigma-Aldrich) for staining the nucleus. Fluorescent images were captured with a TCS SP8 confocal laser scanning microscope (Leica).

### Total RNA isolation and RT–qPCR analysis

The total RNA from cells or tissues was extracted with TRIzol (Thermo Fisher) reagent and was subsequently reverse transcribed to cDNA with PrimeScript RT Master Mix (Takara) according to the manufacturer’s instruction. RT–qPCR was performed with Hieff qPCR SYBR Green Master Mix (Yeasen) on a LightCycler 480 II system. Primers of target gene are listed in Table 1, and β-actin was used as control to normalize expression. Relative expression was calculated with 2^−ΔΔ*CT*^ method.

**Table 1:** Primers for RT–qPCR.

Gene	Forward primer (5′ to 3′)	Reverse prime (5′ to 3′)
*Actb*	GTGACGTTGACATCCGTAAAGA	GCCGGACTCATCGTACTCC
*Psrc1*	TCAAGTTCATTGTGGACGAGAC	GGGAGCTACTTCATTTGGGTT
*Muc2*	CCTTCAACCACCTCACCAACCATC	TCCAGAATCCAGCCAGCCAGTC
*Zo1*	GAAGGCGGATGGTGCTACAAGTG	AGGCTCAGAGGACCGTGTAATGG
*Ocln*	AGTCCACCTCCTTACAGACCTGATG	GCCTCCATAGCCACCTCCGTAG
*Atoh1*	GAGTGGGCTGAGGTAAAAGAGT	GGTCGGTGCTATCCAGGAG
*Hes1*	TCAGCGAGTGCATGAACGAG	CATGGCGTTGATCTGGGTCA
*Lyz1*	GAGACCGAAGCACCGACTATG	CGGTTTTGACATTGTGTTCGC
*Mmp7*	CTTACCTCGGATCGTAGTGGA	CCCCAACTAACCCTCTTGAAGT
*Ido1*	GCCTGCCTCCTATTCTGTCTTATGC	GGGTCCACAAAGTCACGCATCC
*Ido2*	CCGCCTATCCTGGTCCACTCTG	TGTCCTGACTGTGTTGCCGAATG
*Tdo*	TCAGGCTTCCAGAGTCTACAGTTCC	CAAGCAGAGCAGCACCTCCTTC
*Tph1*	CTGCGAAGGAAGACGTTATGGAGAC	TGAGAGGTAACCAGCCACAGGAC
*Kynu*	CCTACTCCAAAGCGGCACAAAATTC	ACCAGCAGGCAAAGTCAACACC
*Kmo*	CGCATGTCAACTCTAGGTGGTTCC	TTCCCATCCAGGCAGGTCTTCTC
*Mao*	CAGGCACAGAGACAGCAACACAG	ACAAAGCAGAGAAGAGCCACAGAAG
*Kat1*	TGGTGACAGTGGGAGCCTATGG	ATCTTGGTGCGAGGTGTGAACTTG
*Kat2*	CACGGTTCCTCACTGCAAC	GCTTTGCTCAGTATGTCCGCT
*Kat3*	CGCATGGATGACCCTGAGTGTTAC	TTGGAGTCGCAGAAGGCAGAAAC
*Ahr*	TGGTTGTCACAGCAGATGCCTTG	GCTCCGTCCTTCCCTTTCTTGTTC
*Cyp1a1*	CAGAAGGTGATGGCAGAGGTTGG	TTAGGCAGGTAACGGAGGACAGG
*Cyp1b1*	GAATCATGACCCAGCCAAGT	TAATGAAGCCGTCCTTGTCC
*ACTB*	CATGTACGTTGCTATCCAGGC	CTCCTTAATGTCACGCACGAT
*PSRC1*	GCCAGCCGAATGCCACTCAC	GTGTCCACTTTCCCGCACTC
*CYP1A1*	ACATGCTGACCCTGGGAAAG	GGTGTGGAGCCAATTCGGAT
*Nppa*	CGTCTTGGCCTTTTGGCTTC	GGTGGTCTAGCAGGTTCTTGAAA
*Nppb*	CTCCAGAGCAATTCAAGATGCAG	CCTACAACAACTTCAGTGCGTTAC
*Myh7*	CTCTGAGCATTCTCCTGCTGT	CTTTCTCGGAGCCACCTTGGA
*cFos*	GGTGAAGACCGTGTCAGGAG	AGTTGATCTGTCTCCGCTTGG

### Determination of tryptophan metabolites in plasma

For the determination of tryptophan metabolites, 50 μl plasma was mixed with 300 μl acetonitrile containing an internal standard, followed by vortex mixing at 1000 rpm for 10 min at 4 °C. Samples were then incubated at −20 °C for 30 min to enhance metabolite extraction efficiency and subsequently centrifugated at 18,000*g*. The supernatant was transferred to new tubes and dried via vacuum centrifugation. Dried residues were reconstituted in 0.1% formic acid aqueous solution with vortexing, then the residual particulates were removed by additional centrifugation. Supernatant was transferred to 96-well plates and maintained at 4 °C until liquid chromatography–mass spectrometry (LC–MS) analysis. Metabolites were analyzed using an ultra-performance liquid chromatography–tandem mass spectrometry (UPLC–MS/MS) system (ACQUITY UPLC-Xevo TQS, Waters Corp.) with a scheduled system. Mobile phase A was 0.1% (v/v) formic acid in ultrapure water and mobile phase B was 0.1% (v/v) formic acid in acetonitrile. The samples were eluted by gradient program as follows: 1% B, 1 min; 10% B, 2 min; 30% B, 4 min; 70% B, 7 min; 99% B, 8.8 min; 99% B, 8.8 min; 1% B, 10 min. The flow rate was 0.40 ml/min. The injection volume was 10.0 μl. Raw UPLC––MS/MS data files were processed using MassLynx Software for metabolites peak detection, integration, calibration and quantitation. Processed datasets were imported into the iMAP (v1.0, Metabo-Profile) metabolomics platform for advanced analysis.

### Cell isolation and culture

Bone marrow-derived macrophages (BMDMs) were isolated from 8–16-week-old male C57BL/6J mice. Following cervical dislocation, mice were immersed in 75% ethanol for sterilizing. After the skin removed, bilateral hindlimbs were transected at coxofemoral and talocrural articulations. The dissected specimens were maintained in PBS containing a 1% penicillin–streptomycin cocktail. The surrounding muscle tissues were removed and the femurs and tibias were transected at both ends. The bone marrow suspension was subsequently collected by flushing with Dulbecco’s modified Eagle medium (DMEM) (Thermo Fisher) containing a 1% penicillin–streptomycin cocktail (Thermo Fisher). The cellular suspension was centrifuged and ultimately reconstituted in complete culture medium containing 10% FBS (ExCell Bio), 1% penicillin–streptomycin cocktail and 20 ng/ml MCSF (GenScript) in DMEM. Cells were cultured at 37 °C in a 5% CO_2_-humidified incubator, with complete medium replacement conducted at 48-h intervals. Following 7-day in vitro differentiation, BMDMs were prepared for subsequent experiments.

Primary murine peritoneal macrophages (MPM) were obtained from *Psrc1*^+/+^*Apoe*^−/−^and *Psrc1*^−^^/−^*Apoe*^−/−^ mice a fed with HFD. Peritoneal cells were collected from mice by sterile lavage and subsequently cultured in complete medium containing 10% FBS and a 1% penicillin–streptomycin cocktail in DMEM for 6–8 h. Following this incubation period, adherent cells were collected as MPM.

Human peripheral blood mononuclear cells (PBMCs) were isolated from blood samples using Ficoll-Paque Premium (17544203, Cytiva) solution-based gradient centrifugation, which differentially separates blood components according to their densities. PBMCs were washed in PBS and cultured in complete medium containing 10% FBS and 1% penicillin–streptomycin cocktail in RPMI 1640 (Thermo Fisher).

The Raw264.7 cell line was obtained from the American Type Culture Collection (ATCC). Raw264.7 was detached with culture medium, followed by centrifugation and resuspension in fresh complete medium containing 10% FBS in DMEM at 48-h intervals. MAEC was obtained from the China Center for Type Culture Collection. The MOVAS cell line was obtained from ATCC and was cultured in DMEM supplemented with 10% FBS. Cells were routinely subcultured at 3-day intervals through 0.25% trypsin–EDTA detachment for refreshing culture medium.

### Determination of IAA

IAA levels in plasma samples were determined via the IAA ELISA kit (ELK7852, ELK Biotechnology) according to the manufacturer’s instructions. Standards and the samples were incubated with a biotinylated-conjugate solution for 60 min at 37 °C. The plate was washed and incubated with streptavidin–HRP for 1 h at 37 °C, followed by washing and incubating with TMB for 20 min. Stop reagent was added into plates and optical density (OD) was measured at 450 nm on an ELISA plate reader. The IAA concentrations were calculated as standard curve based on the OD of the serial dilution of IAA.

### Cell transfection

The plasmid (CMV-MCS-3FLAG-SV40-EGFP) carrying the full-length coding of the mouse *Psrc1* gene was subcloned into adenovirus vector as in our previous study^8^. BMDMs were infected with adenovirus vector control (AdGFP) or Psrc1 (Ad*Psrc1*) for 10 h. After 10 h of infection, the cells were refreshed with complete culture medium for 48–72 h to conduct subsequent experiments. To knock down *Psrc1* or *cFos*, BMDMs were transfected with si*Psrc1* using Lipofectamine RNAiMAX (Thermo Fisher) in OptiMEM (Thermo Fisher) for 6 h. After 6 h of transfection, the medium was replaced with complete culture medium. Following 48–72 h transfection, cells were collected or used in subsequent experiments. The sequences of siCtrl, si*Psrc1* and si*cFos* are listed in Table 2.

**Table 2:** Sequences for RNA silencing.

Gene	Antisense (5′ to 3′)	Sense (5′ to 3′)
siCtrl	ACGUGACACGUUCGGAGAATT	UUCUCCGAACGUGUCACGUTT
si*Psrc1*	UCUGGACUCACUAAUACUGTT	CAGUAUUAGUGAGUCCAGATT
si*cFos*	UCAUCUUCAAGUUGAUCUGTT	CAGAUCAACUUGAAGAUGATT

### Bacterial strains and culture conditions

*A. muciniphila* (ATCC-BAA-835) was obtained from ATCC and cultured in brain–heart infusion (Thermo Fisher) supplemented with 0.5% type II mucin (Sigma-Aldrich) and 0.05% L-cysteine (Sigma-Aldrich) at 37 °C under anaerobic conditions. *A. muciniphila* was suspended in PBS at 1 × 10^9^ c.f.u./ml via oral gavage administration to mice. The heat-killed *A. muciniphila* was heated at 121 °C for 15 min.

### Total protein extraction and western blot analysis

Cell samples were lysed with RIPA buffer supplemented with a 1% protease inhibitor cocktail. The protein concentration was quantified with the BCA Protein Assay Kit (Yeasen). Equivalent protein samples were electrophoresed in SDS–PAGE gel and subsequently transferred to PVDF membranes (Millipore). Membranes were blocked in 5% skimmed milk for 1–2 h at room temperature and incubated with primary antibodies against Psrc1 (GTX128047, GeneTex), Ahr (67785-1-Ig, Proteintech), cleaved caspase 3 (9661S, CST), Bax (2772S, CST), Uchl3 (8141S, CST), phospho-P38 (4511 T, CST), total P38 (8690 T, CST), phospho-Jnk (4668 T, CST), total Jnk (66210-1-Ig, Proteintech), phospho-Erk1/2 (9101 T, CST), total Erk1/2 (9102 T, CST), Ubiquitin (3936S, CST), cFos (66590-1-Ig, Proteintech), α-Tubulin (80762-1-RR, Proteintech) and Lamin B (66095-1-Ig, Proteintech) at 4 °C overnight. Following incubation with anti-mouse or rabbit HRP-conjugated IgG secondary antibodies (SA00001-1/SA00001-2, Proteintech). The blotting bands were detected using an ECL system.

### Nuclear and cytoplasmic extraction

Nuclear and cytoplasmic proteins were extracted using the NE-PER Nuclear Cytoplasmic Extraction Reagent kit (Thermo Fisher) according to the manufacturer’s protocol. BMDMs were lysed with CER I supplemented with protease inhibitor cocktail at 4 °C. After thorough vortexing to ensure uniform lysis, samples were incubated on ice for 10 min. CER II was added and samples were vortexed intermittently followed by a 1-min ice incubation. The lysates were then centrifuged at 16,000*g* for 5 min at 4 °C. The cytoplasmic protein in supernatant was collected. The insoluble fraction was resuspended using NER supplemented with protease inhibitor cocktail. Samples were incubated for 40 min with vigorous vortexing for 15 s every 10 min to facilitate nuclear protein release. Following incubation, samples were centrifuged at 16,000*g* for 10 min at 4 °C. Nuclear protein was collected in the supernatant.

### Co-immunoprecipitation

Cells were washed with PBS and lysed in 10% SDS for 10 min. Protein samples were boiled for 5 min, supplemented with IP buffer (Beyotime Biotechnology) containing protease inhibitor and then centrifuged at 14,000 rpm. One out of ten samples were maintained at −80 °C as input controls, and the remaining supernatant was incubated with mouse IgG (ab18421, Abcam) or anti-Ahr antibody (sc133088, Santa Cruz Biotechnology) via mild rotation at 4 °C overnight. The magnetic beads were washed with IP buffer and incubated with protein samples containing antibody for 4 h with rotation. The magnetic beads (B23201, Selleck Chemicals) absorbing the antigen–antibody complex were magnetically isolated and washed with PBST three times. The precipitated proteins were eluted using 1× SDS–PAGE loading buffer and boiled for 10 min. The precipitated and input proteins were analyzed using western blotting.

### Determination of ALT and CRE

Alanine aminotransferase (ALT) and creatinine (CRE) levels in serum samples were determined via the Alanine Aminotransferase Assay kit (Nanjing JianCheng Bioengineering Institute) and the Creatinine Assay kit (Nanjing JianCheng Bioengineering Institute) according to the manufacturer’s instructions.

### Quantification and statistical analysis

All the experimental data are presented as mean ± s.d., and no outliers were excluded in this study. SPSS 29.0 was adopted for the statistical analysis. The normal distribution of the data was determined by the Shapiro–Wilk normality test. The statistical comparisons of normally distributed variables between two groups were performed using two-tailed unpaired *t*-tests, while the non-normally distributed variables were compared using the Mann–Whitney *U* test. The normally distributed variables of more than two groups were analyzed using one-way analysis of variance (ANOVA) or two-way ANOVA followed by Tukey’s or Tamhane’s T2 post hoc test, while non-normally distributed variables were analyzed using the Kruskal–Wallis *H* test. Rank-based inverse normal transformation of PSRC1 relative expression level, IAA level and CYP1A1 relative expression were performed, and then partial Pearson correlation coefficients were performed. The *P* values are shown in the figures and *P* < 0.05 was considered statistically significant.

## Results

### Psrc1 deficiency accelerates atherosclerotic lesion and reduces colonic mucus layer in vivo

**Fig. 1:**
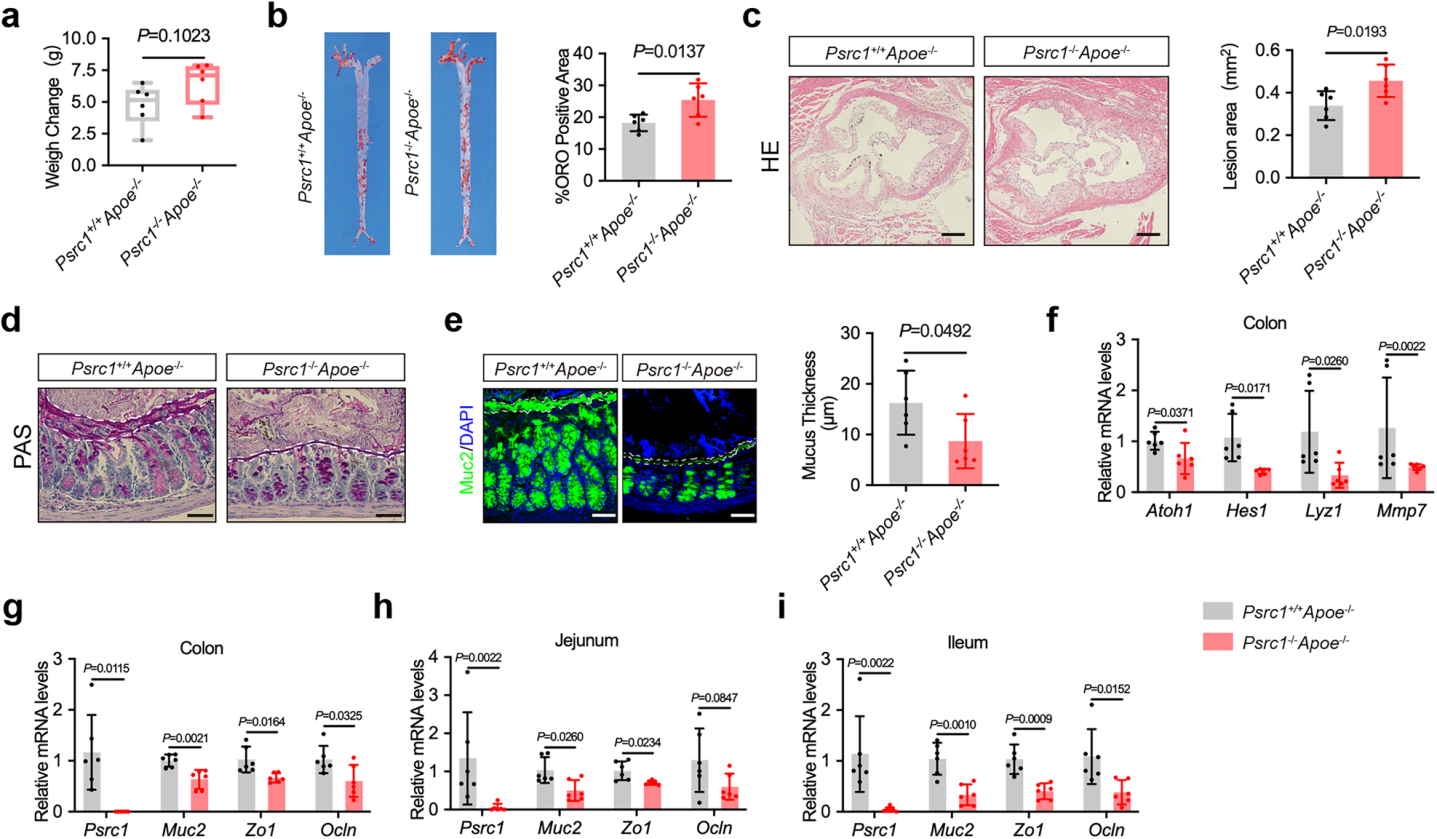
Psrc1 deficiency accelerates atherosclerotic lesion and reduces colonic mucus layer in vivo*.* Eight-week-old male *Psrc1*^+/+^*Apoe*^−/−^ and *Psrc1*^−/−^*Apoe*^−/−^ mice were fed a HFD for 12 weeks (*n* = 6). **a** The weight change was determined post- HFD versus pre-diet baseline. **b** Representative images of ORO staining and quantitative analysis of the atherosclerotic plaques area en face aorta. **c** Representative photomicrograph of HE staining and quantification of atherosclerotic plaques area of aortic roots. Scale bar, 200 μm. **d** Representative photomicrograph of Periodic Acid-Schiff stain (PAS) staining of colonic sections. Scale bar, 50 μm. **e** Representative images of immunofluorescence staining of Muc2 protein and DAPI for nuclei in colonic sections and the quantification of fluorescence positive mucus thickness. Scale bar, 50 μm. **f** The relative mRNA levels of *Atoh1*, *Hes1*, *Lyz1* and *Mmp7* (to *β-actin*) in colon were determined using RT–qPCR. **g–i** The relative mRNA levels of *Psrc1*, *Muc2*, *Zo1* and *Ocln* (to *β-actin*) in colon (**g**), jejunum (**h**) and ileum (**i**) determined using RT–qPCR. Data are presented as mean ± s.d. The *P* values were determined by two-tailed unpaired *t-*tests (**a**–**c** and **e**; *Atoh1* and *Hes1* in **f** and **g**; *Zo1* and *Olcn* in **h**; and *Muc2*, *Zo1* and *Ocln* in **i**) or Mann–Whitney *U* test (*Lyz1* and *Mmp7* in **f**, *Psrc1* and *Muc2* in **h** and *Psrc1* in **i**).

Host metabolic enzymes and gut microbiota synergistically regulate Trp metabolism, with *A. muciniphila* emerging as a key modulator of Trp catabolic pathways^18,33,34^. To explore the role of Psrc1 deficiency in atherogenesis, *Psrc1*^+/+^*Apoe*^−/−^ and *Psrc1*^−/−^*Apoe*^−/−^ mice were fed a HFD for 12 weeks. Consistent with our previous research, *Psrc1*^−/−^*Apoe*^−/−^ was notably increased compared to *Psrc1*^+/+^*Apoe*^−/−^ (Fig. 1a). In agreement with our previous research, *Psrc1*^−/−^*Apoe*^−/−^ mice exhibited significantly increased atherosclerotic lesion burden in both the aorta en face and aortic root compared to *Psrc1*^+/+^*Apoe*^−/−^ controls (Fig. 1b,c). As a well-characterized mucin-degrading bacterium, *A. muciniphila* preferentially colonizes the colonic mucosal layer, utilizing host-derived mucin glycoproteins as its primary nutrient substrate. To investigate the symbiotic relationship, the mucus layer and Muc2, the most abundant mucin in colon, were detected. The mucus thickness was notably declined in *Psrc1*^−/−^*Apoe*^−/−^ mice compared to *Psrc1*^+/+^*Apoe*^−/−^ (Fig. 1d,e). Goblet and Paneth cells serve as the predominant secretory lineages. Compared to controls, *Psrc1*^−/−^*Apoe*^−/−^ mice showed significantly decreased colonic mRNA levels of *Atoh1* and *Hes1*, which were involved in goblet cell maturation, as well as *Lyz1* and *Mmp7*, which were markers of Paneth cells (Fig. 1f). Moreover, the structural integrity of the mucus layer and intestinal epithelial barrier is fundamental to preserving gut homeostasis. *Muc2* mRNA, which is a marker of the mucus layer, and *Zo1* and *Ocln* mRNA, which are markers of the tight junction, showed significant decreases in the colon, jejunum and ileum of *Psrc1*^−/−^*Apoe*^−/−^ compared to *Psrc1*^+/+^*Apoe*^−/−^ (Fig. 1g–i).

Taken together, these findings indicate that in the atherosclerotic model, Psrc1 deficiency reduces mucus layer and impairs intestinal barrier function, which may explain the declined *A. muciniphila* abundance.

### Psrc1 has an effect on Trp metabolism in atherosclerosis

**Fig. 2:**
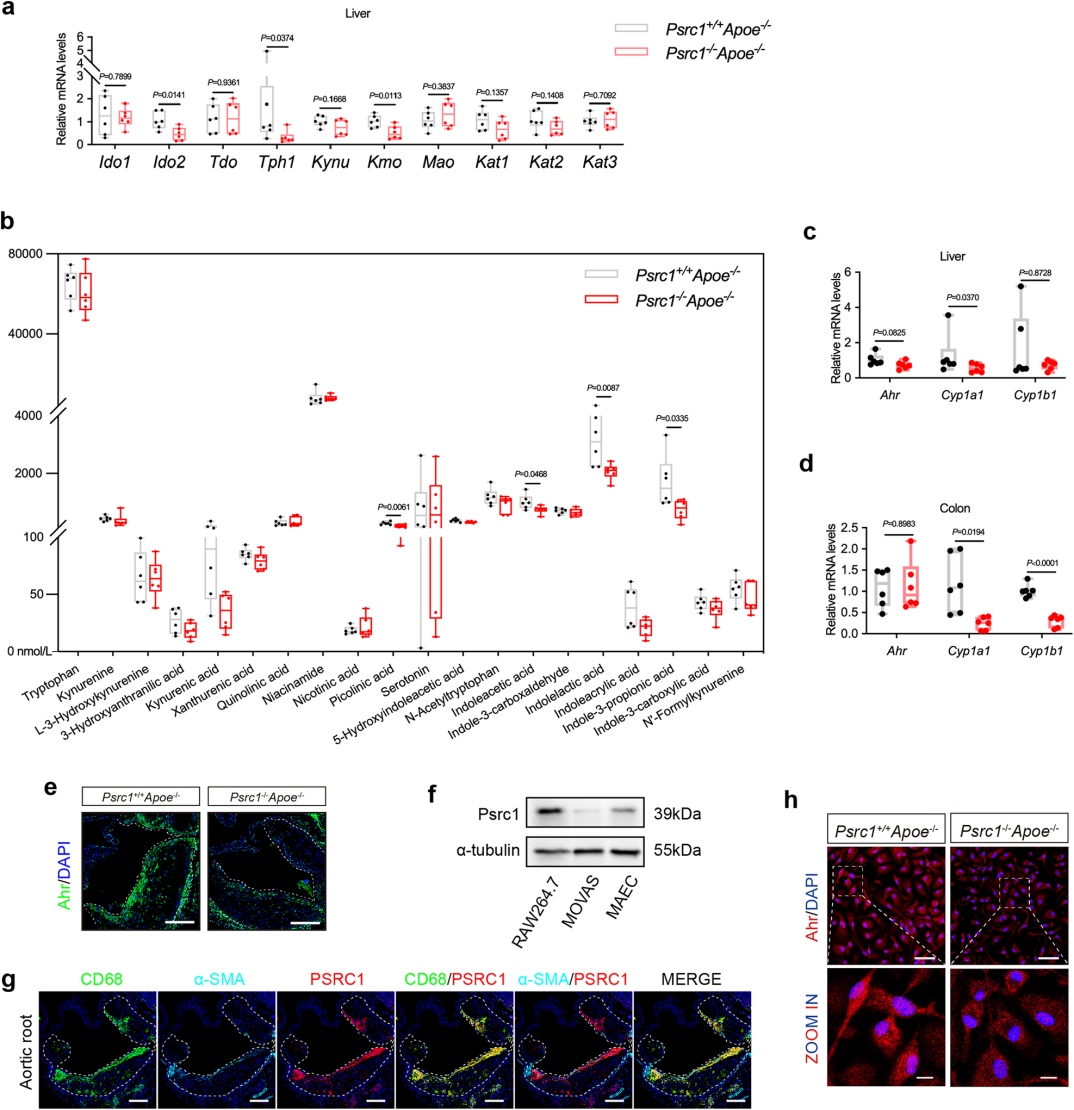
Psrc1 has an effect on Trp metabolism in atherosclerosis. **a** The relative mRNA levels of essential Trp metabolic enzymes (to *β-actin*) in liver were determined using RT–qPCR (*n* = 6). **b** The Trp metabolites in plasma (*n* = 6). **c**,**d** The relative mRNA levels of *Ahr*, *Cyp1a1* and *Cyp1b1* (to *β-actin*) in liver (**c**) and colon (**d**) were determined using RT–qPCR (*n* = 6). **e** Immunofluorescence of aortic root cross-sections demonstrating staining for Ahr in plaques. Scale bar, 200 μm. **f** Psrc1 protein expression in Raw264.7, MOVAS and MAEC determined by western blotting. **g** Immunofluorescence of aortic root cross-sections demonstrating staining for Psrc1 in CD68^+^ macrophages and α-SMA^+^ smooth muscle cells. Scale bar, 150 μm. **h** Representative images of immunofluorescence staining of Ahr protein and DAPI for nuclei in MPM isolated from *Psrc1*^+/+^*Apoe*^−/−^ and *Psrc1*^−/−^*Apoe*^−/−^ mice. Scale bar, 50 μm (top) and 10 μm (bottom). Data are presented as mean ± s.d. Statistical analysis was performed by two-tailed unpaired *t-*tests (*Ido1*, *Ido2*, *Kynu*, *Kmo*, *Mao*, *Kat1*, *Kat2* and *Kat3* in **a** and **b** and *Ahr* in **c** and **d**) and Mann–Whitney *U* test (*Tdo* and *Tph1* in **a** and *Cyp1a1* and *Cyp1b1* in **c**).

The essential Trp metabolic enzymes in liver and circulating Trp metabolites were determined. *Ido2*, *Tph1*and *Kmo* mRNA levels, which are essential Trp metabolic enzymes, were reduced in *Psrc1*^−/−^*Apoe*^−/−^ mice liver compared to *Psrc1*^+/+^*Apoe*^−/−^ (Fig. 2a). Interestingly, picolinic acid, IAA, ILA and IPA levels also were markedly decreased in *Psrc1*^−/−^*Apoe*^−/−^ mice plasma (Fig. 2b). IAA, ILA and IPA are derived from Trp via the indole pathway mediated by intestinal microbial communities. In addition, Ahr serves as the predominant receptor of indole derivatives, with its activation driving the expression of target genes *Cyp1a1* and *Cyp1b1*^37^. To evaluate whether Psrc1 deficiency impacts AhR pathway activity, we analyzed the expression of AhR and its downstream targets. In the liver, *Ahr* and *Cyp1b1* mRNA levels remained unchanged, while *Cyp1a1* mRNA levels were significantly decreased in *Psrc1*^−/−^*Apoe*^−/−^ mice (Fig. 2c). In the colon, *Ahr* mRNA levels were comparable between the two groups, but both *Cyp1a1* and *Cyp1b1* mRNA levels were reduced in *Psrc1*^−/−^*Apoe*^−/−^ mice (Fig. 2d). Ahr expression in plaques of aortic roots were also reduced in *Psrc1*^−/−^*Apoe*^−/−^ mice relative to *Psrc1*^+/+^*Apoe*^−/−^ (Fig. 2e), providing further evidence for decreased Trp metabolites levels.

To investigate the subcellular localization of Psrc1, we analyzed its expression profile across key atherosclerotic plaque components: macrophages, vascular smooth muscle cells and endothelial cells. Psrc1 expression was substantial higher in Raw264.7 compared to MOVAS and MAEC (Fig. 2f). In addition, macrophages and smooth muscle cells were the predominant origins contributing to foamy cells. Immunofluorescence staining of aortic root plaques further demonstrated robust colocalization of Psrc1 with the macrophage-specific marker CD68, with minimal overlap with the vascular smooth muscle cell marker α-SMA (Fig. 2g). These results suggest that Psrc1 was predominantly expressed within macrophage populations, which supports our previous observation of colocalization of Psrc1 with F4/80-positive macrophages^8^. Furthermore, murine peritoneal macrophages were confirmed via CD68 immunofluorescence staining (Supplementary Fig. 1a). Consistently, reduced accumulation of Ahr was observed in peritoneal macrophages from *Psrc1*^−/−^*Apoe*^−/−^ mice compared to *Psrc1*^+/+^*Apoe*^−/−^ (Fig. 2h). Taken together, these results suggest Psrc1 participates in regulating Trp metabolism.

### IAA level is reduced in patients with CAD

**Fig. 3:**
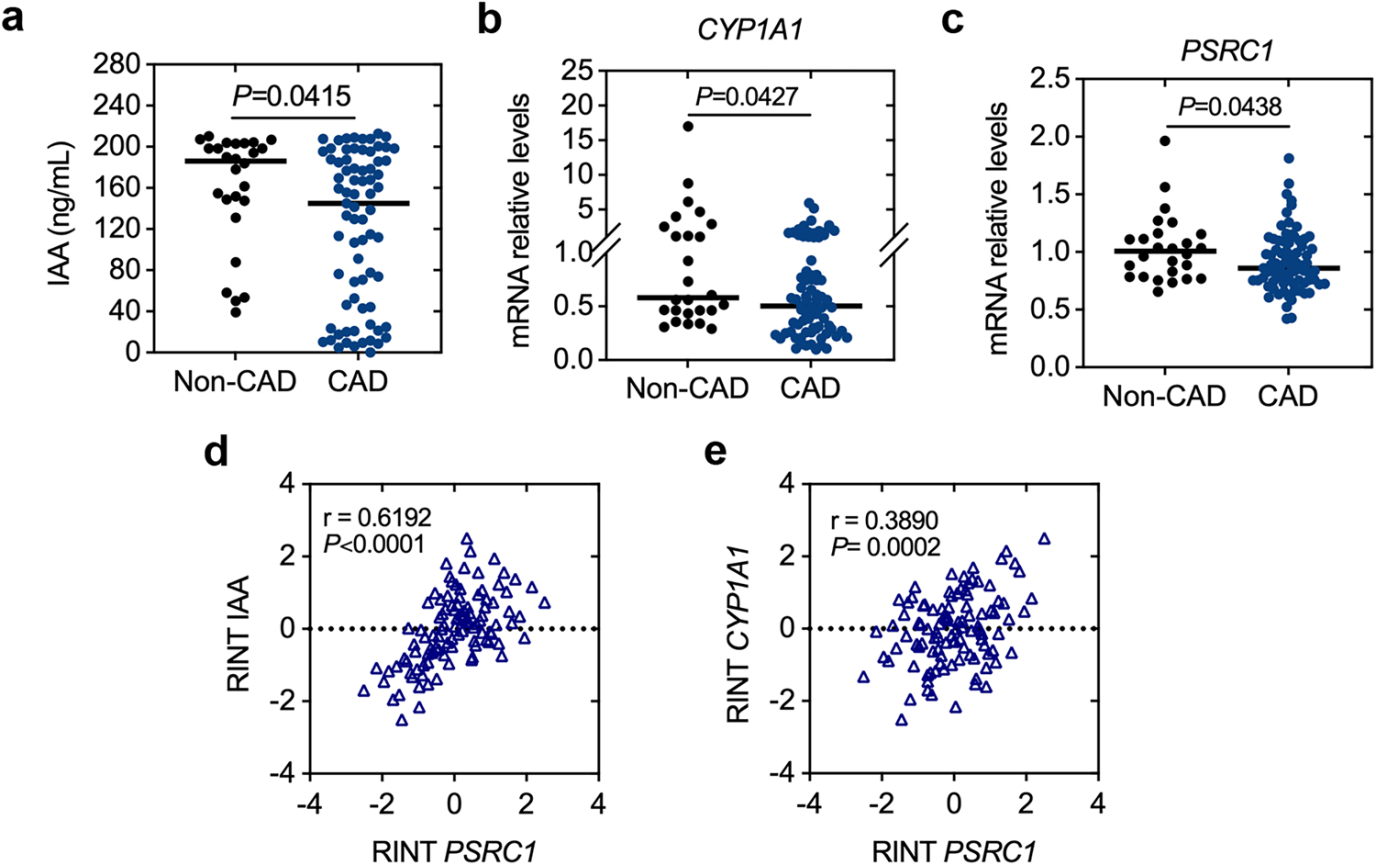
IAA level is reduced in patients with CAD. **a** The plasma IAA levels in patients without (non-CAD) and with CAD determined by ELISA. **b**,**c** The relative mRNA levels of *CYP1A1* (**b**) and *PSRC1* (**c**) (to *β-actin*) in PBMCs isolated from non-CAD and patients with CAD determined using RT–qPCR (*n* = 26 in in the non-CAD group and *n* = 77 in the CAD group). **d**, A rank-based inverse normal transformation of IAA levels and relative mRNA levels of *PSRC1* were performed and the correlation between rank-based inverse IAA levels (RINT IAA) and *PSRC1* expression (RINT *PSRC1*) was analyzed in non-CAD and patients with CAD (*n* = 103). **e** Rank-based inverse normal transformation of relative mRNA levels of *CYP1A1* were performed and the correlation between rank-based inverse *CYP1A1* expression (RINT *CYP1A1*) and *PSRC1* expression (RINT *PSRC1*) was analyzed in non-CAD and patients with CAD (*n* = 103). Data are presented as mean ± s.d. Statistical analysis was performed by a Mann–Whitney *U* test (**a–c**). Correlation analysis was performed by the partial Pearson test (**d** and **e**).

In vitro studies have demonstrated that *A. muciniphila* can metabolize tryptophan to produce IAA, which in turn enhances the proliferative capacity of *A. muciniphila* in a feedback loop. In vivo studies using a colitis model have consistently revealed that *A. muciniphila* colonization potentiates intestinal IAA biosynthesis. Mechanistically, IAA exerts protective effects against atherosclerosis via anti-inflammatory activity, attenuation of oxidative stress, inhibition of apoptosis and modulation of metabolic homeostasis. Notably, our previous study identified *A. muciniphila* as the most substantially differentially abundant microorganism following *Psrc1* gene knockout, providing a compelling rationale for selecting IAA as the primary investigative target in subsequent explorations. Baseline information was collected from patients with and without CAD on demographic characteristics, medical history and lipid-lowering medication use (Supplementary Table 1). To validate the reduced IAA levels observed in the atherosclerosis model, we quantified plasma IAA levels and *CYP1A1* mRNA in circulating PBMCs from patients with and without CAD. Consistent with our murine findings, IAA levels were lower in CAD (Fig. 3a). Concomitantly, *CYP1A1* mRNA levels were also markedly reduced in PBMCs from patients with CAD (Fig. 3b), supporting a potential causal link between diminished IAA signaling and CAD pathogenesis. Moreover, reduced *PSRC1* mRNA levels were also observed in CAD PBMCs (Fig. 3c), consistent with our previous studies. A correlation analysis reveals that the rank-based inverse normal transformed PBMC *PSRC1* levels were positively correlated with the IAA and *CYP1A1* levels (Fig. 3d,e), further suggesting that PSRC1 regulates IAA production. Collectively, these results suggest Psrc1 participates in regulating Trp metabolism and Psrc1 deficiency may reduce IAA production in atherosclerosis.

### Psrc1 upregulates Ahr expression through attenuation of ubiquitination-mediated degradation in macrophages

**Fig. 4:**
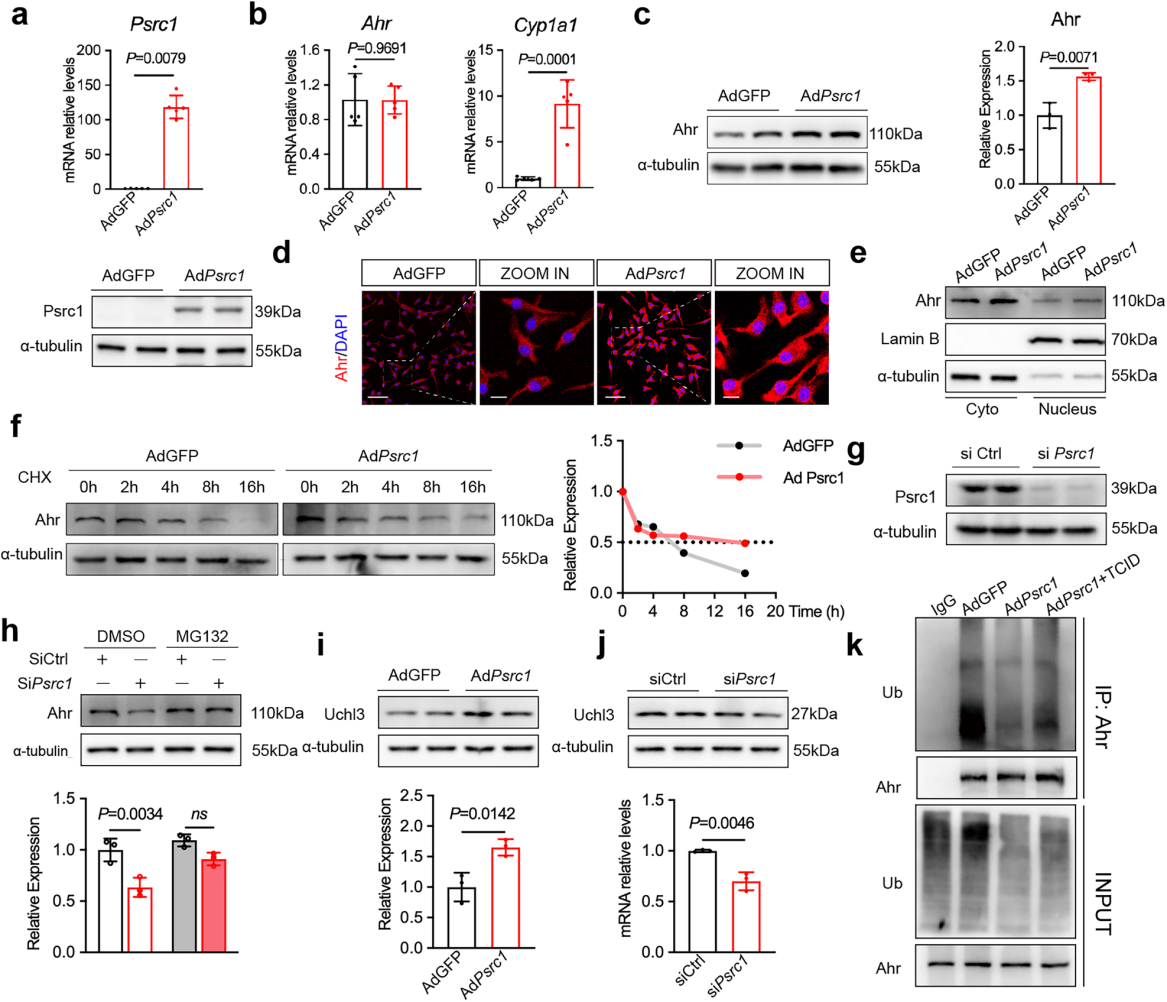
Psrc1 upregulates Ahr expression through attenuation of ubiquitination-mediated degradation in macrophages. **a** *Psrc1* mRNA and protein expression levels in were determined using RT–qPCR (top) and western blotting (bottom). **b** *Ahr* and *Cyp1a1* mRNA expression levels were determined using RT–qPCR (*n* = 5). **c** Ahr protein expression was determined using western blotting. Two duplicate lanes were used as technical repetitions (*n* = 3). **d** Representative images of immunofluorescence staining of Ahr protein and DAPI for nuclei (*n* = 6). **e** Ahr protein expression in the cytoplasmic and nuclear fraction was determined using western blotting (*n* = 3). **f** After the treatment with CHX (15 μg/ml) for the indicated durations, Ahr protein expression was analyzed by western blotting. **g** Psrc1 protein expression levels were determined using western blotting. **h** BMDMs were treated with siCtrl or si*Psrc1* for 48 h followed by DMSO or MG132 (10 μM) treatment for 6 h. Ahr protein expression levels were determined using western blotting (*n* = 3). **i**,**j** Uchl3 protein expression levels in BMDMs treated with Ad*Psrc1* (**i**) or si*Psrc1* (**j**) were determined using western blotting (*n* = 3). **k** BMDMs treated with AdGFP or Ad*Psrc1* for 48 h followed by DMSO or TCID (10 μM) treatment for 24 h and MG132 treatment for 6 h before immunoprecipitation of Ahr and western blot of ubiquitin. Data are presented as mean ± s.d. Statistical analysis was performed by two-tailed unpaired *t-*tests (**b**, **c**, **i** and **j**), Mann–Whitney *U* test (**a**) and two-way ANOVA followed by Tukey’s post hoc test (**h**). BMDMs were treated with AdGFP or Ad*Psrc1* for 72 h in **a**–**f** and **i**. BMDMs were treated with siCtrl or si*Psrc1* for 48 h in **g**, **h** and **j**.

Our preceding results demonstrated the reduced expression of Ahr in Psrc1-deficient mice. While in vivo physiological conditions suggest that Psrc1 may indirectly regulate Ahr via modulation of Trp metabolite levels, here we will specifically explore the direct regulatory mechanism by which Psrc1 influences Ahr. To this end, we employed BMDMs as an in vitro model to eliminate confounding factors from systemic metabolism and gut microbiota, thereby enabling a focused investigation of the direct interactions between Psrc1 and Ahr. The cells isolated from bone marrow were stained with F4/80 and were confirmed as BMDMs (Supplementary Fig. 1b). Increased *Cyp1a1* but not *Ahr* mRNA levels were seen in BMDMs transfected with Ad*Psrc1* (Fig. 4a,b), consistent with our aforementioned findings in vivo, while western blot and immunofluorescence staining showed elevated Ahr protein levels in BMDMs transfected with Ad*Psrc1* (Fig. 4c,d). In addition, as a ligand-activated transcription factor, Ahr undergoes nuclear translocation upon activation. Therefore, we assessed Ahr protein levels in both cytoplasmic and nuclear fraction. Western blot of the fraction revealed increased Ahr levels were both seen in cytoplasmic and nuclear fraction of BMDMs transfected with Ad*Psrc1* (Fig. 4e). These results suggest the influence of Psrc1 on Ahr protein abundance was not attributed to changes in *Ahr* transcription. Previous research indicated that ubiquitin carboxyl terminal hydrolase L3 (Uchl3) could stabilize Ahr in non-small-cell lung cancers^38^. To investigate whether Psrc1 regulates Ahr protein stability through the deubiquitylation pathway, BMDMs transfected with AdGFP or Ad*Psrc1* were subjected to cycloheximide (CHX) chase treatment, followed by quantitative analysis of Ahr protein degradation rate. The Ahr protein half-life was prolonged when Psrc1 was overexpressed in BMDMs (Fig. 4f). Consistently, BMDMs with siRNA-mediated Psrc1 knockdown exhibited a reduction in Ahr protein levels. Notably, cotreatment with the proteasome inhibitor MG132 effectively rescued the Psrc1 knockdown-induced downregulation of Ahr protein (Fig. 4g,h). These findings collectively indicate that Psrc1 deficiency promotes Ahr degradation via the ubiquitin–proteasome system, highlighting a post-translational regulatory mechanism by which Psrc1 stabilizes the Ahr protein. To test the hypothesis that Psrc1 regulated Ahr stability via Uchl3-mediated deubiquitylation, we evaluated the effect of Psrc1 overexpression and knockdown on Uchl3. Increased Uchl3 was seen in BMDMs transfected with Ad*Psrc1*, whereas decreased Uchl3 in BMDMs transfected with si*Psrc1* (Fig. 4i,j). Building upon existing evidence that cFos transcriptionally upregulates Uchl3^39^, we hypothesize that Psrc1 modulates Uchl3 through regulation of cFos expression. Transfection with Ad*Psrc1* enhanced cFos expression (Supplementary Fig. 2a,b), whereas siRNA-mediated *Psrc1* knockdown reduced cFos expression (Supplementary Fig. 2c,d). Subsequently, siRNA-mediated cFos knockdown led to a reduction in Uchl3 (Supplementary Fig. 2e,f). Intriguingly, Psrc1 overexpression failed to upregulate Uchl3 under cFos-knockdown conditions (Supplementary Fig. 2g). These data established cFos as the essential downstream mediator of Psrc1-driven Uchl3 regulation. The immunoprecipitation assays revealed that overexpression of *Psrc1* decreased the ubiquitinated form of Ahr compared to control, while such a decrease was attenuated by the Uchl3 inhibitor TCID (Fig. 4k). Taken together, these data demonstrated that Psrc1 suppresses Ahr ubiquitination proteasome degradation mediated by Uchl3, leading to elevated protein levels of Ahr in BMDMs.

### Oral administration of live *A. muciniphila* rescues Psrc1 deficiency-mediated aggravated atherosclerosis and declined IAA level

**Fig. 5:**
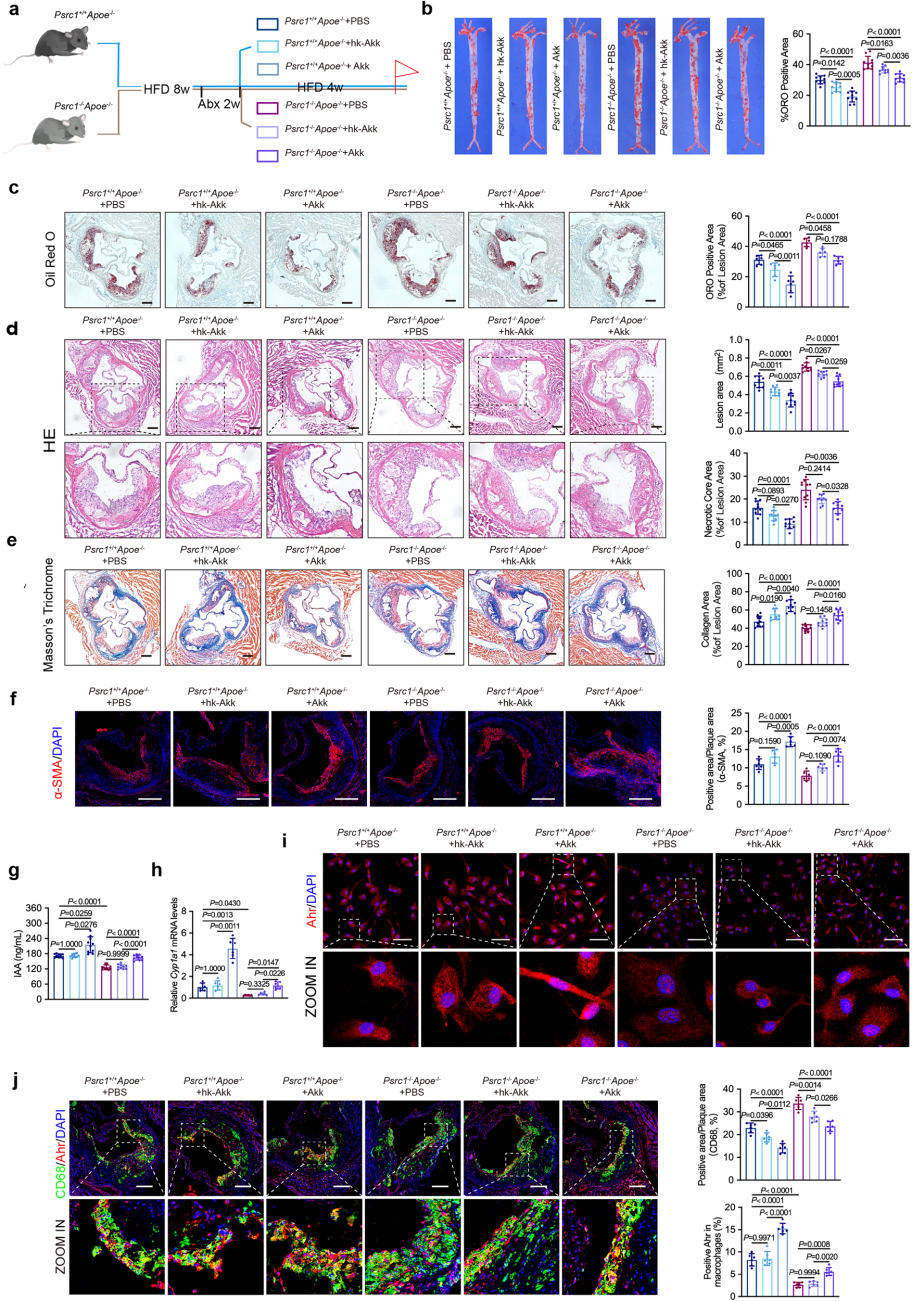
Oral administration of live *A. muciniphila* rescues Psrc1 deficiency-mediated aggravated atherosclerosis and reduced IAA. **a** The experimental design showing groups and durations. Eight-week-old male *Psrc1*^+/+^*Apoe*^−/−^ and *Psrc1*^−/−^*Apoe*^−/−^ mice fed a HFD for 12 weeks. Mice were orally gavaged with antibiotic cocktail for 2 weeks and subsequently with live *A. muciniphila* strain or heat-killed *A. muciniphila* or PBS for 4 weeks during the last 6 weeks. **b** Representative images of ORO staining and quantitative analysis of the atherosclerotic plaques area en face aorta (*n* = 10). **c–e** Representative photomicrograph of ORO (**c**), HE (**d**) and Masson’s trichrome (**e**) staining and quantification of lipid accumulation, atherosclerotic plaques area, necrotic core and collagen content in aortic roots (*n* = 10). Scale bar, 200 μm. **f** Immunofluorescence of aortic root cross-sections demonstrating staining α-SMA for smooth muscle cells and DAPI for nuclei (*n* = 6). Scale bar, 200 μm. **g** The plasma IAA levels were determined by ELISA (*n* = 10). **h** The relative mRNA levels of *Cyp1a1* (to *β-actin*) in MPM isolated from mice were determined using RT–qPCR (*n* = 6). **i** Representative images of immunofluorescence staining of Ahr protein and DAPI for nuclei in MPM isolated from mice (*n* = 6). Scale bar, 50 μm. **j** Immunofluorescence of aortic root cross-sections demonstrating staining CD68 and Ahr to detect macrophage infiltration and Ahr in macrophages (*n* = 6). Scale bar, 200 μm. Data are presented as mean ± s.d. *P* values were determined by two-way ANOVA followed by Tukey’s post hoc test (**b**, c, **d** (top), **e**, **f** and **j**) or Tamhane’s T2 post hoc test (**d** (bottom), **g** and **h**).

*A. muciniphila* abundance was predominantly decreased in Psrc1-deficient mice. To investigate whether administration of live *A. muciniphila* can mitigate Psrc1 deficiency-induced atherosclerotic exacerbation and restore reduced IAA level, *Psrc1*^+/+^*Apoe*^−/−^ and *Psrc1*^−/−^*Apoe*^−/−^ mice were fed with HFD for 12 weeks. The experimental design included a 2-week pretreatment with an oral antibiotic cocktail to deplete endogenous gut microbiota, followed by a 4-week intervention phase where mice received daily oral gavage of either live *A. muciniphila*, heat-killed *A. muciniphila* or vehicle (Fig. 5a).

Oral gavage administration of *A. muciniphila* reduced the weight and atherosclerotic plaque area of aorta in *Psrc1*^+/+^*Apoe*^−/−^ and *Psrc1*^−/−^*Apoe*^−/−^ mice (Supplementary Figs. 3a and Fig. 5b). In addition, an increase in lipid content, lesion area and necrotic core area and a decrease in collagen area induced by deficiency of Psrc1 were attenuated by *A. muciniphila* supplementation (Fig. 5c–e). The decreased infiltration of smooth muscle cells and increased macrophages caused by Psrc1 deficiency were also reversed by oral administration of live *A. muciniphila* (Fig. 5f,j). Although both live and heat-killed *A. muciniphila* demonstrated potential in alleviating atherosclerosis in *Psrc1*^+/+^*Apoe*^−/−^ and *Psrc1*^−/−^*Apoe*^−/−^ mice, it is important to highlight that live *A. muciniphila* exhibited more pronounced protective properties, suggesting that the observed protective effect is probably primarily dependent on microbial metabolites rather than the bacterial components of *A. muciniphila*.

Moreover, the declined plasma IAA levels and *Cyp1a1* mRNA in peritoneal macrophages resulting from Psrc1 deficiency were abrogated with live *A. muciniphila* administration to a certain extent (Fig. 5g,h). Importantly, Ahr in macrophages of plaques and peritoneal macrophages were decreased in *Psrc1*^−/−^*Apoe*^−/−^ mice compared to *Psrc1*^+/+^*Apoe*^−/−^ administrated with PBS, which were rescued by supplementation with live *A. muciniphila* (Fig. 5i,j). However, heat-killed *A. muciniphila* failed to restore IAA levels, *Cyp1a1* mRNA and Ahr expression in macrophages. Taken together, oral administration of live *A. muciniphila* ameliorates Psrc1 deficiency-induced atherosclerotic exacerbation while restoring diminished IAA levels. These findings not only validate live *A. muciniphila* as a promising therapeutic candidate for targeting Psrc1-mediated pathways but also identifies IAA as a critical downstream mediator through which Psrc1 exerts its atheroprotective effects.

### IAA mitigates Psrc1 knockdown-mediated apoptosis in macrophages

**Fig. 6:**
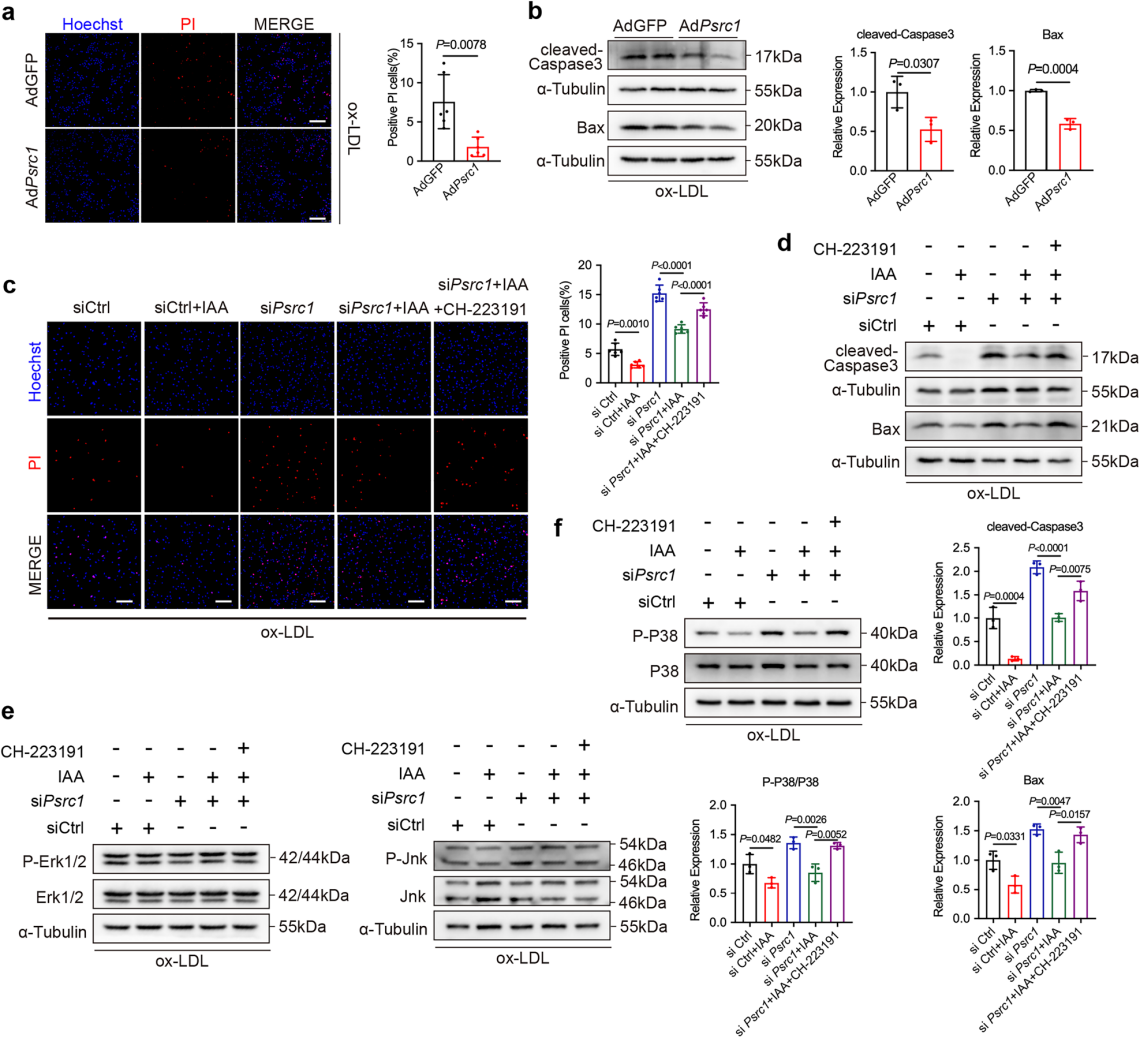
IAA mitigates Psrc1 knockdown-mediated apoptosis in macrophages. **a** Immunofluorescence of staining Hoechst (blue) and PI (red) to detect cell death (*n* = 6). Scale bar, 100 μm. **b** Bax and cleaved-Caspase3 protein expression were determined using western blotting. Two duplicate lanes were used as technical repetitions (*n* = 3). **c** Immunofluorescence of staining Hoechst (blue) and PI (red) to detect cell death (*n* = 6). Scale bar, 100 μm. **d** Bax and cleaved-Caspase3 protein expression were determined using western blotting (*n* = 3). **e**, **f** P-Erk1/2, Erk1/2, P-Jnk, Jnk (**e**), P-P38 and P38 (**f**) protein expression levels were determined using western blotting (*n* = 3). BMDMs were treated with AdGFP or Ad*Psrc1* for 72 h, followed by stimulation with 100 μg/ml ox-LDL in the last 48 h in **a** and **b**. BMDMs were treated with siCtrl or si*Psrc1*, followed by stimulation with DMSO or IAA (10 μM) individually or IAA with CH-223191 (10 μM) for 24 h and subsequently treated with 100 μg/ml ox-LDL in the last 48 h in **c–f**. Data are presented as mean ± s.d. Statistical analysis was performed by two-tailed unpaired *t-*tests (**a** and **b**) and one-way ANOVA followed by Tukey’s post hoc test (**c**, **d** and **f**).

Emerging studies have highlighted the pivotal contribution of macrophage apoptosis in driving atherosclerotic plaque progression and promoting the formation and expansion of necrotic cores^2^. Psrc1 has been implicated in the regulation of mitotic cell division and proliferative processes, with prior evidence identifying it as a downstream target of p53^40^, which serves as a pivotal regulator involved in cell cycle and cell death pathways. Its potential role in apoptotic modulation remains undetermined. BMDMs were transfected with either AdGFP or Ad*Psrc1*. Subsequently, cells were treated with ox-LDL to evaluate the regulatory effect of Psrc1 on macrophage apoptosis. Hoechst and Propidium Iodide (PI) staining showed a decreased percentage of death cells in BMDMs overexpressing *Psrc1* (Fig. 6a). Concomitantly, reduced cleaved-Caspase3 and Bax, which is highly expressed in apoptotic cells, were observed in BMDMs transfected with Ad*Psrc1* compared to AdGFP (Fig. 6b). These results suggest Psrc1 inhibits apoptosis in macrophages.

The role of IAA in apoptosis remains controversial. Pro-apoptotic effects were observed in both HL-60 and NSO cell lines. Paradoxically, contrasting evidence revealed that IAA exhibited anti-apoptotic activity in macrophages. We therefore examined whether IAA could mitigate the exacerbated macrophage apoptosis induced by Psrc1 knockdown and further explored whether this protective effect was mediated via the Ahr pathway using the specific Ahr antagonist CH-223191. IAA treatment reduced the percentage of dead cells and alleviated the elevated apoptotic rate caused by Psrc1 knockdown, but this protective effect of IAA was substantially attenuated by cotreatment with CH-223191 (Fig. 6c). In addition, increased cleaved-Caspase3 and Bax were seen in BMDMs treated with si*Psrc1* compared to control, which were rescued via treatment of IAA. However, the rescue effect of IAA was abolished by CH-223191 (Fig. 6d). The MAPK pathway is involved in cell apoptosis, which was subsequently determined using western blot. The ratios of P-Erk1/2 to total Erk1/2 and P-Jnk to total Jnk remained unchanged among groups. By contrast, the ratio of P-P38 to total P38 was increased in BMDMs treated with si*Psrc1*. IAA treatment reduced the ratio of P-P38 to total P38 induced by si*Psrc1*. Critically, this inhibitory effect of IAA was abrogated by cotreatment with CH-223191 (Fig. 6e,f). Taken together, these results suggest IAA mitigates the aggravated apoptosis induced by Psrc1 knockdown via the Ahr/P38 pathway in macrophages.

### Oral administration of IAA rescues aggravated atherosclerosis caused by Psrc1 deficiency via alleviating macrophages apoptosis

**Fig. 7:**
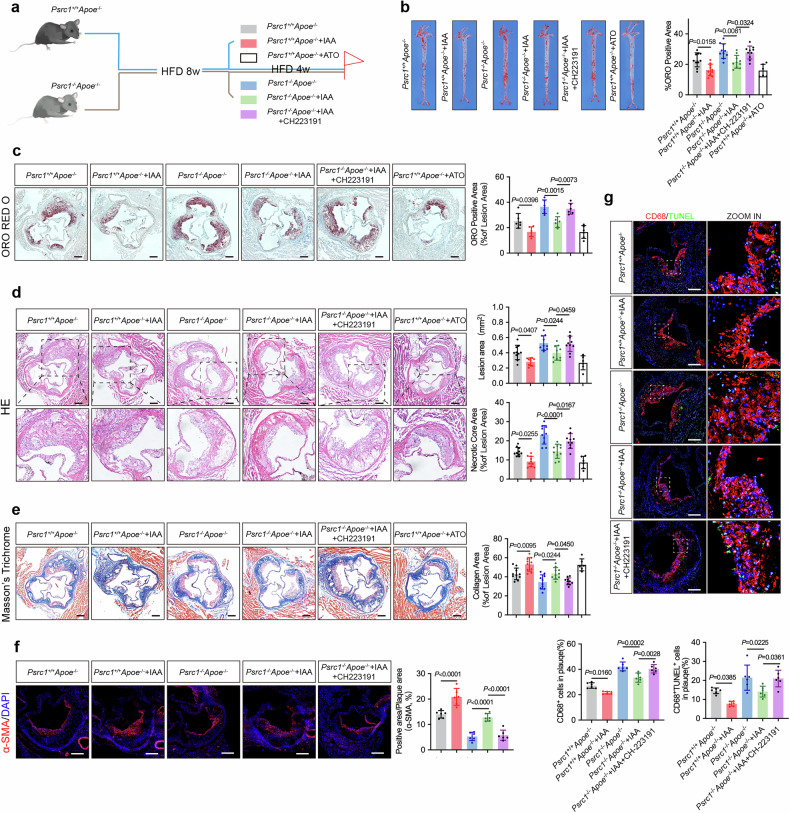
Oral administration of IAA rescues aggravated atherosclerosis caused by Psrc1 deficiency via alleviating macrophages apoptosis. **a** The experimental design showing groups and durations. Eight-week-old male *Psrc1*^+/+^*Apoe*^−/−^ and *Psrc1*^−/−^*Apoe*^−/−^ mice fed a HFD for 12 weeks. *Psrc1*^+/+^*Apoe*^−/−^ mice were orally administrated with vehicle or IAA (50 mg/kg/day) or atorvastatin (15 mg/kg/day), while *Psrc1*^−/−^*Apoe*^−^^/^^−^ were orally administrated with vehicle or IAA individually or IAA with CH-223191 (10 mg/kg/day) during the last 4 weeks. **b** Representative images of ORO staining and quantitative analysis of the atherosclerotic plaques area en face aorta (*n* = 6 in the atorvastatin group, *n* = 10 in other groups). **c–e** Representative photomicrograph of ORO (**c**), HE (**d**) and Masson’s trichrome (**e**) staining and quantification of lipid accumulation, atherosclerotic plaques area, necrotic core and collagen content in aortic roots (*n* = 6 in the atorvastatin group, *n* = 10 in other groups). Scale bar, 200 μm. **f** Immunofluorescence of aortic root cross-sections demonstrating staining α-SMA for smooth muscle cells and DAPI for nuclei (*n* = 6). Scale bar, 200 μm. **g** Immunofluorescence of aortic root cross-sections demonstrating staining for CD68 and TUNEL to detect macrophage infiltration and apoptotic macrophages (*n* = 6). Scale bar, 200 μm. Data are presented as mean ± s.d. *P* values were determined by one-way ANOVA followed by Tukey’s post hoc test.

IAA was elucidated as the ‘mediator’ of *A. muciniphila* in an atherosclerotic model. It was demonstrated that IAA could mitigate the exacerbated apoptosis induced by *Psrc1* knockdown. To detect the effect of IAA in vivo, following HFD feeding for 12 weeks, *Psrc1*^+/+^*Apoe*^−/−^ were orally administrated with vehicle or IAA or atorvastatin, and *Psrc1*^−/−^*Apoe*^−/−^ mice were orally administrated with vehicle or IAA solely or IAA accompanied by CH-223191 in the last 4 weeks (Fig. 7a). Oral administration of IAA reduced the atherosclerotic plaque area en face aorta in *Psrc1*^+/+^*Apoe*^−/−^, while IAA administration also reduced the increased atherosclerosis plaque area caused by Psrc1 deficiency, which was simultaneously abrogated by intervention with CH-223191 (Fig. 7b). Moreover, decreased cross-sectional lipid accumulation, lesion area and necrotic core area and increased collagen content were observed in *Psrc1*^+/+^*Apoe*^−/−^ treated with IAA. Notably, these atheroprotective effects of IAA were also displayed in *Psrc1*^−/−^*Apoe*^−/−^ mice, which was reversed via using CH-223191 (Fig. 7c–e). In addition, the increased filtration of smooth muscle cells and decreased macrophages were seen in *Psrc1*^+/+^*Apoe*^−/−^ and *Psrc1*^−/−^*Apoe*^−/−^ mice administrated with IAA, whereas the effect from IAA in *Psrc1*^−/−^*Apoe*^−/−^ mice was abolished with CH-223191 (Fig. 7f,g). Further, decreased apoptotic macrophages in plaques were observed in *Psrc1*^+/+^*Apoe*^−/−^ administrated with IAA when the increased apoptotic macrophages mediated by Psrc1 deficiency was reduced by treatment with IAA. However, this difference in *Psrc1*^−/−^*Apoe*^−/−^ mice was abrogated via simultaneous CH-223191 treatment (Fig. 7g). Moreover, no hepatic or kidney dysfunction was detected in *Psrc1*^+/+^*Apoe*^−/−^ and *Psrc1*^−/−^*Apoe*^−/−^ mice (Supplementary Fig. 4a,b). Taken together, these findings suggest that IAA exerts Psrc1-mediated atheroprotection by reducing macrophage apoptosis through activation of the Ahr pathway.

Furthermore, we also systematically evaluated the dose-dependent toxicological profile of IAA through comprehensive physiological monitoring. Oral administration of 500 mg/kg IAA elicited marked hematotoxicity, evidenced by reduction in red blood cell counts and hemoglobin, while white blood cell (WBC) and platelet counts remained stable (Supplementary Fig. 5a–d). Cardiac hypertrophic biomarkers showed pathological elevation with upregulation of *Nppa*, *Nppb* and *Myh7* mRNA levels (Supplementary Fig. 6a–c). ALT and CRE both exhibited notable elevation when mice were orally gavaged with 500 mg/kg IAA (Supplementary Fig. 7a,b). Importantly, the atherosclerosis-protective 50 mg/kg dose exhibited no detectable adverse effects across these parameters, establishing its therapeutic safety.

## Discussion

Our previous study revealed the role of Psrc1 in modulating gut microbiota and metabolites to alleviate atherosclerosis. The current study demonstrated Trp metabolism was reprogrammed in Psrc1-deficient mice, whose plasma QA, IAA, ILA and IPA levels were markedly decreased. Especially, IAA levels in polasma from patients with CAD were decreased and showed a positive correlation with *PSRC1* mRNA in PBMCs. On the one hand, Ahr, the receptor of IAA, was downregulated in Psrc1-deficient mice, contributing to the alteration in Trp metabolites in vivo. On the other hand, the Ahr protein was stabilized in *Psrc1*-overexpressing macrophages, resulting from increased Uchl3-mediated deubiquitylation. In addition, oral administration of live *A. muciniphila* led to the recovery of plasma IAA levels and atherosclerosis in *Psrc1*^−/−^*Apoe*^−/−^ mice. Moreover, IAA supplementation in *Psrc1*^−/−^*Apoe*^−/−^ mice brought about an anti-atherosclerotic effect via reducing apoptosis of macrophages in plaques in vivo, which was abolished via using the Ahr inhibitor CH-223191. This study identified the ‘Psrc1–*A. muciniphila*–IAA–Ahr axis’ as a candidate target of atheroprotection.

We found the mucus layer and Muc2 in *Psrc1*^−/−^*Apoe*^−/−^ mice colons were decreased, in tandem with decreased expression of the markers of colonic secretory lineage, such as *Atoh1*, *Hes1*, *Lyz1* and *Mmp7*. As a mucin-degrading specialist, *A. muciniphila* derives its nomenclature from its mucinophilic property^41^. It not only utilizes intestinal mucin glycoproteins as nutrient sources but also maintains intestinal homeostasis through dynamic interaction with the mucus layer. *A. muciniphila* stimulate mucin secretion and regulate mucin degradation, which explains the decreased abundance of *A. muciniphila* in Psrc1-deficient mice. Our data also demonstrated that decreased *Muc2*, *Zo1* and *Ocln* mRNA were observed in Psrc1-deficient mice intestine. This indicates the impaired mucus barrier and mechanical barrier in Psrc1-deficient mice. In alignment with a previous study^14^, our results showed that *A. muciniphila* led to a reduction in the circulating endotoxin levels in *Apoe*^−/−^ mice fed a HFD via regulating Zo1 and Ocln. Mounting studies have revealed that dysfunctional intestinal barrier integrity is a pivotal component in inflammatory bowel disease^42–44^. Dysfunctional mucus and goblets cells always precede inflammation, which indicate its role in the inflammatory pathogenesis^45^. In addition, our previous study demonstrated that *Psrc1* knockout resulted in a proinflammatory colonic phenotype^8^, which may cause the impaired intestinal barrier function. These findings together support the potential role of Psrc1 in modulating the mucus layer to maintain intestinal homeostasis and regulate *A. muciniphila* abundance.

It was demonstrated that Psrc1 was involved in reprogramming Trp metabolism, including the alteration in host metabolic enzymes of liver and the metabolites levels in plasma. Interestingly, the marked change in production was primarily concentrated in the indole pathway, including IAA, ILA and IPA. The indole pathway was largely completed through contributions from the intestinal microbiota^30^. Nonetheless, the potential role of *A. muciniphila* in catabolizing Trp has been identified in vitro. Trp can be catabolized into indole, IAA and ILA, and these products promoted the growth of *A. muciniphila* in reverse^33^. Furthermore, indole and its derivatives are involved in inflammatory responses, lipid metabolism, oxidative stress and programmed cell death^20,21,46–48^, which play essential roles in atherosclerosis. It has been demonstrated that IPA inhibits atherosclerosis via promoting reverse cholesterol transport in macrophages and IPA was reduced in patients with CAD^20^. ILA led to the activation of Ahr to mitigate colitis. It can also activate the Ahr/Nrf2 pathway to enhance hepatocellular antioxidant capacity, thus alleviating liver injury^49^. In addition, IAA alleviates the inflammation response and free radicals in Raw264.7^22^. In a nonalcoholic fatty liver disease model, IAA attenuated inflammatory and oxidative stress and hepatic lipogenesis^21^. A recent study showed that IAA, as the metabolite of *Bacteroides ovatus*, alleviated atherosclerosis via inhibiting the TLR4/MyD88/NF-κB pathway to promote M2 macrophage polarization and reduce aortic inflammation^50^. In agreement with our findings, the IAA level was also reduced in patients with CAD. Furthermore, our data demonstrated the positive correlation between Psrc1 and IAA levels in patients, thus indicating the potential role of Psrc1 in producing IAA. However, another study showed that *A. muciniphila* could notably offset the IAA levels in colitis models^34^. Taking all these findings together, Psrc1 mitigates atherosclerosis via not only promoting reverse cholesterol transport but also manipulating gut microbiota and its metabolites. Hence, Trp metabolites, in particular IAA, are part of the mediating effects of Psrc1 in alleviating atherosclerosis, whereas the correlation of *A. muciniphila* and IAA in patients with CAD was not elucidated in our study.

The Ahr protein was degraded via ubiquitination. We found Ahr was not only downregulated in Psrc1-deficient mice, attributed to its regulation in Trp metabolites in vivo, but also decreased in Psrc1-knockdown macrophages independent of Trp metabolites in vitro. The Ahr protein was stabilized via Uchl3-mediated deubiquitylation in non-small-cell lung cancers^38^, in line with our findings in BMDMs. In addition, we found that Uchl3 was upregulated by overexpressing Psrc1 in macrophages. Furthermore, a previous study demonstrated that Uchl3 expression was regulated by cFos in gastric cancer stem-like cells^39^. Our current study also validated that Psrc1 modulated Uchl3 via regulating cFos in BMDMs.

Oral administration of live *A. muciniphila*, rather than heat killed, was found to markedly mitigate atherosclerosis in our study and recover IAA levels in Psrc1-deficient mice. Therefore, the atherosclerosis-protective effect on Psrc1-deficient mice was mediated by the metabolic property of *A. muciniphila* in our current study. Li et al. used an 8-week Western diet-induced atherosclerotic model to confirm that oral administration of live or heat-killed *A. muciniphila* decreased the atherosclerotic plaque area via alleviation of the inflammation responses^14^. We used a 12-week HFD-induced atherosclerotic model and oral administration of *A. muciniphila* in the last 4 weeks following an antibiotic cocktail gavage, which demonstrated that *A. muciniphila* not only decrease the plaque area but also stabilized the plaque in the aortic root. This indicates that these effects could be attributed to the recovery of IAA levels, to some extent. Several types of intestinal microbiota including *Lactiplantibacillus plantarum*, *Bacteroides ovatus*, *Lactobacillus reuteri, Acinetobacter radioresistens* and *A. muciniphila* have been reported capable of metabolizing Trp to IAA^50–53^.

Apoptotic macrophages or foamy cells in plaques play a pivotal role in atherosclerosis. The accumulation of apoptotic cells in plaques indicate greater instability^2^. Psrc1 is involved in regulating cell mitosis and cell cycle and is the target of P53. Psrc1 dose-dependently inhibited ASPP2 in a P53-mediated Bax promoter activation^54^, indicating the anti-apoptotic potential of Psrc1. Consistently, our data suggest the anti-apoptotic function of Psrc1 in macrophages treated with ox-LDL. Exposure to IAA caused cellular apoptosis in zebrafish embryos^55^, whereas microbiome-derived IAA in the airway alleviated apoptosis via macrophage–epithelial cell crosstalk to reduce lung function decline^56^. Therefore, the effect of IAA on cell apoptosis shows conflicting results. However, we found IAA could reduce the apoptosis mediated by Psrc1 knockdown, whereas the effect was abolished by an Ahr inhibitor. PM2.5 induced melanogenesis via Ahr/MAPK signaling activation^57^. Low–medium polarity ginsenosides from wild ginseng alleviated intestinal disturbance via restoring IAA and IPA levels to activate the Ahr/MAPK pathway^58^. These findings together unveiled that the MAPK pathway mediates the signal transduction of Ahr. In addition, it was demonstrated that IAA reduced macrophage apoptosis via the Ahr/MAPK P38 pathway in our study. However, only decreased phosphorylated P38 but not Erk1/2 and Jnk was observed in macrophages treated with IAA, which may be attributed to the various cell types and treatment models.

Furthermore, our data showed the anti-atherosclerotic effect of 50 mg/kg IAA in Psrc1-deficient mice without hepatic and kidney dysfunction. In this regard, a recent study by Liu et al. also found IAA, as the metabolite of *Bacteroides ovatus*, improved atherosclerosis via inhibiting inflammation^50^. We elucidated that the atheroprotective effect mediated by IAA is attributable to the protection of plaque macrophages from apoptosis. However, 500 mg/kg IAA exposure to rats for 14 days caused hematotoxicity and toxic effects in several tissues^59^ and 500 mg/kg IAA in rats induced marked cardiac stress^28^. In addition, we 500 mg/kg IAA induced hematotoxicity, cardiotoxicity, hepatic and renal toxicity. Red blood cell and hemoglobin counts were markedly declined, while WBC and platelet counts did not exhibit any alteration. Intriguingly, in the graded concentration range of 10–250 mg/kg, oral administration of IAA moderately reduced WBC counts, which may contribute to its anti-inflammatory properties. Importantly, the absence of significant changes in WBC counts at 500 mg/kg compared to controls does not preclude possible effects. It has been demonstrated that 500 mg/kg IAA induced cardiac hypertrophy in Wistar albino rats^28^. *Nppa*, *Nppb* and *Myh7* are hypertrophic markers^60^ and our study found that these markers were elevated in *Apoe*^−/−^ mice orally administered 500 mg/kg IAA, suggesting this dose of IAA treatment may have a role in pathological cardiac remodeling and cause heart hypertrophy. That said, the 50 mg/kg IAA used in our current research was safe to mitigate atherosclerosis, while 500 mg/kg exhibited marked side effects.

The administration of an Ahr inhibitor in Psrc1-deficient mice abolished this protection. These findings collectively revealed the atheroprotective functions of Ahr. Conversely, TCDD led to Ahr-dependent development of atherosclerosis via the induction of macrophage inflammation in the vasculature^61^. However, Kim et al. found that Ahr in smooth muscle cells maintained lesion cap integrity in atherosclerotic tissues^62^. On the one hand, our Ahr-targeting inhibitor was administered orally and acted across multiple tissues; therefore, contributions from nonvascular tissues to atherosclerotic progression cannot be excluded. On the other hand, the opposing effects of Ahr reported across studies may be partly attributable to differences in the ligands used to activate Ahr. Accordingly, investigations employing diverse Ahr ligands or cell type-specific Ahr targeting via in vivo gene editing are required to clarify the role of Ahr in atherosclerosis.

In conclusion, our study reported that Psrc1 exerts atheroprotective effects, which are mediated by *A. muciniphila* and IAA. Deficiency of Psrc1 leads to declined *A. muciniphila* abundance and IAA levels, ultimately aggravating atherosclerosis via exacerbated macrophages apoptosis. This study highlights the potential of *A. muciniphila* and IAA as dual therapeutic targets for both prevention and treatment for patients with CAD with PSRC1 loss-of-function variants.

## Supplementary information

Supplementary Information
