# Supplementary material for: Proline/serine-rich coiled-coil 1 alleviates atherosclerosis via remodeling tryptophan metabolism mediated by Akkermansia muciniphila
Source: Exp Mol Med. 2026 Mar 6;58(3):848–63. doi: 10.1038/s12276-026-01668-5 (PMC13049064; doi:10.1038/s12276-026-01668-5)
Supplement: Supplementary file 1 — Supplementary Information [file 12276_2026_1668_MOESM1_ESM.pdf]

Supplementary figure legends

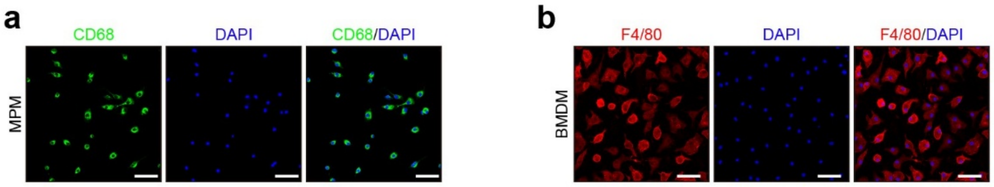

Supplementary Fig. 1. The validation of primary macrophages. a Immunofluorescence staining of CD68 and DAPI for nuclei in murine peritoneal macrophages (MPMs) isolated from *Psrc1*$^{+/+}$*Apoe*$^{-/-}$ mice. Scale bar, 50 μm. **b** Immunofluorescence staining of F4/80 and DAPI for nuclei in bone marrow-derived macrophages (BMDMs) isolated from C57BL/6J mice. Scale bar, 50 μm.

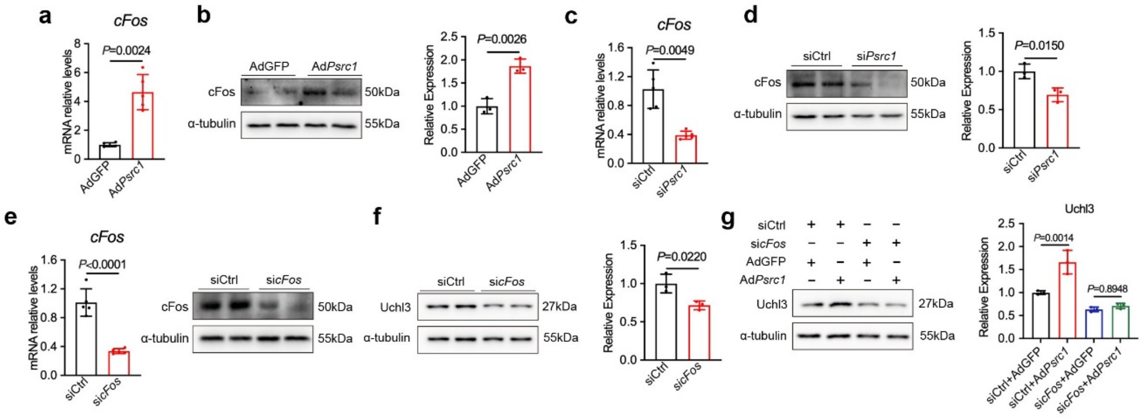

Supplementary Fig. 2. Psrc1 upregulates Uchl3 via modulating cFos expression in BMDMs. BMDMs were treated with AdGFP or Ad*Psrc1* for 72 h in **a** and **b**. **a**, **b** *cFos* mRNA and protein expression levels were determined using qRT-PCR (**a**) (*n*=5) and Western blotting (**b**) (*n*=3). BMDMs were treated with siCtrl or si*Psrc1* for 72 h in **c** and **d**. **c**, **d** *cFos* mRNA and protein expression levels were determined using qRT-PCR (**a**) (*n*=5) and Western blotting

(**b**) (*n*=3). BMDMs were treated with siCtrl or si*Psrc1* for 72 h in **e** and **f**, **e** *cFos* mRNA and protein expression levels were determined using qRT-PCR (left) and Western blotting (right). **f** Uchl3 protein expression levels were determined using Western blotting (*n*=3). **g** BMDMs were treated with siCtrl or si*cFos* for 72 h followed by AdGFP or Ad*Psrc1* treatment during the last 48 hours. Uchl3 protein expression levels were determined using Western blotting (*n*=3). Data are presented as mean ± SD. Statistical analysis was performed by two tailed unpaired *t* test (**a**-**f**) and two-way ANOVA followed by Tukey post hoc test (**g**).

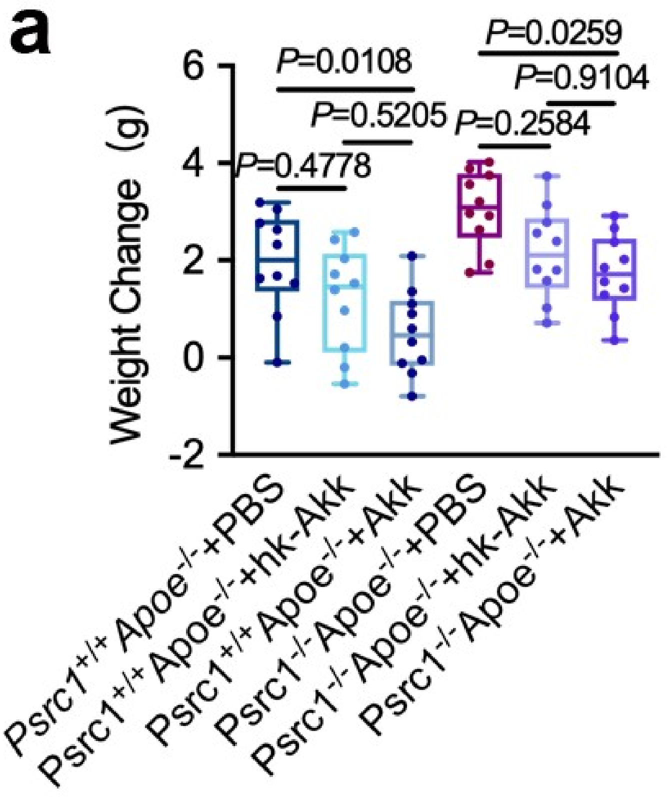

Supplementary Fig. 3. Oral administration of *A.muciniphila* decreases Psrc1-deficiency-mediated weight change increase. Mice were grouped and treated as Fig. 5. **a** The weight

change was determined post *A.muciniphila* or PBS gavage versus pre-gavage baseline.

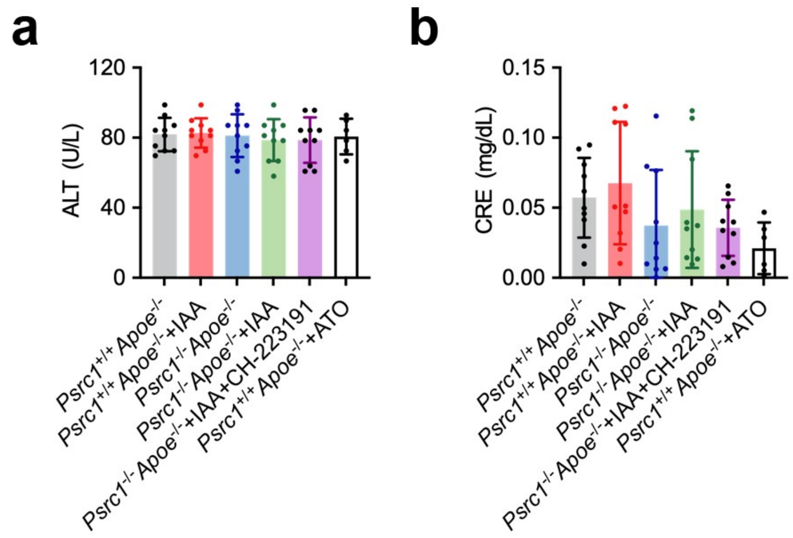

Supplementary Fig. 4. No hepatic and kidney dysfunction were observed in mice treated with IAA. Mice were grouped and treated as Fig. 7 (n=10). **a, b** The hepatic function and kidney function were respectively determined using ALT and CRE levels in serum.

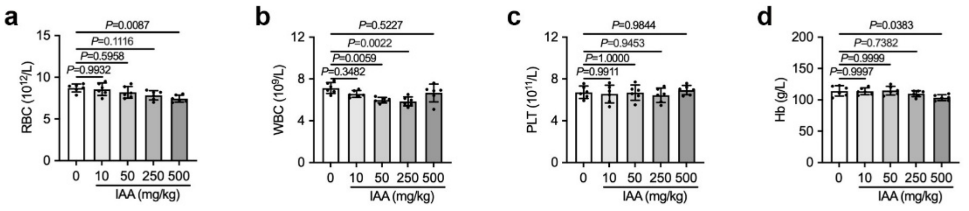

Supplementary Fig. 5. Excessive dose of IAA induced hematotoxicity. IAA was orally gavaged at gradient doses (10, 50 250, 500 mg/kg) to 16-week-old male *Apoe*$^{-/-}$ mice (n=6 in each groups). **a-d** Counts of peripheral blood cells including RBC (**a**), WBC (**b**), PLT (**c**) and hemoglobin (**d**) were detected. Data are presented in as mean ± SD. Statistical analysis was

performed by one-way ANOVA followed by the Tukey post hoc test.

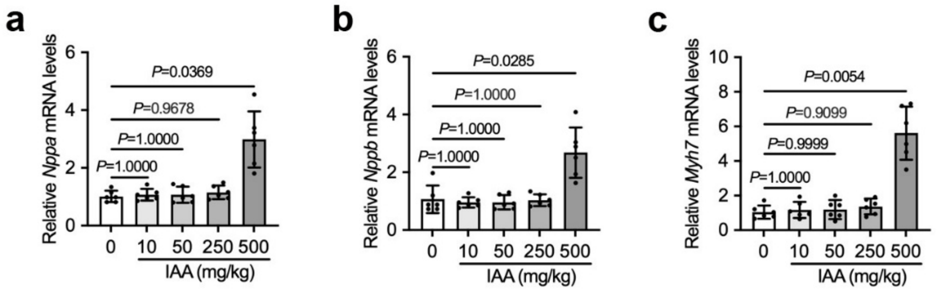

Supplementary Fig. 6. Excessive dose of IAA induced cardiotoxicity. *Apoe*$^{-/-}$ mice were grouped and treated as Supplementary Fig. 5. **a-c** The relative mRNA levels of *Nppa* (**a**), *Nppb* (**b**) and *Myh7* (**c**) (to *β-actin*) in cardiac tissues were determined using qRT-PCR (*n*=6). Data are presented as mean ± SD. Statistical analysis was performed by one-way ANOVA followed by the Tamhane's T2 post hoc test.

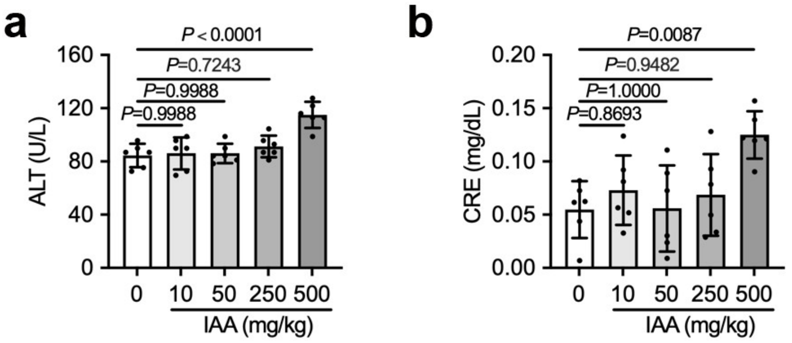

Supplementary Fig. 7. Excessive dose of IAA induced hepatic and kidney dysfunction. *Apoe*$^{-/-}$ mice were grouped and treated as Supplementary Fig. 5. **a, b** The hepatic function and kidney function were respectively determined using ALT (**a**) and CRE (**b**) levels in serum. Data

are presented as mean ± SD. Statistical analysis was performed by one-way ANOVA followed by the Tukey post hoc test.

Supplementary Table 1: Baseline characteristics according to the patients with or without CAD

Characteristics	Non-CAD (*n*=26)	CAD (*n*=77)	*P* value
Age, years	56 ± 7.60	59.75 ± 9.15	0.0626
Male, No. (%)	14 (53.8)	53 (68.8)	0.1659
BMI, kg/m^2	23.72 ± 3.14	25.44 ± 3.07	0.0152
Current smoking, No. (%)	17 (65.4)	52 (67.5)	0.8404
Hypertension, No. (%)	16 (61.5)	58 (75.3)	0.1766
Diabetes mellitus, No. (%)	16 (61.5)	50 (64.9)	0.7550
Total cholesterol, mmol/L	4.73 ± 0.75	4.98 ± 1.24	0.3294
LDL cholesterol, mmol/L	2.72 ± 0.71	2.83 ± 1.13	0.6438
HDL cholesterol, mmol/L	1.27 (1.14-1.43)	1.06 (0.93-1.27)	0.0004
Triglycerides, mmol/L	1.69 (1.31-2.96)	2.31 (1.27-3.04)	0.3000
Lipid-lowering medication usage, No. (%)	20 (76.9)	69 (89.6)	0.1027
CREA (μmol/L)	72.15 ± 13.61	78.21 ± 16.34	0.0923
ALT (U/L)	16.5 (13.75-21.25)	19 (15-25)	0.2637
AST (U/L)	18 (15.75-22.25)	20 (17-24)	0.2135

Mean (±SD) values, median (IQR) values and percentage (%) are shown.
ALT, alanine aminotransferase; AST, aspartate aminotransferase; BMI, body mass index; CAD, coronary artery disease; CREA, creatine; HDL, high density lipoprotein; IQR, interquartile range; LDL, low density lipoprotein; SD, standard deviation; TC, Total cholesterol; TG, Triglycerides.
